# Larval Environment Alters Amphibian Immune Defenses Differentially across Life Stages and Populations

**DOI:** 10.1371/journal.pone.0130383

**Published:** 2015-06-24

**Authors:** Katherine L. Krynak, David J. Burke, Michael F. Benard

**Affiliations:** 1 Department of Biology, Case Western Reserve University, Cleveland, Ohio, United States of America; 2 Research Department, The Holden Arboretum, Kirtland, Ohio, United States of America; Ecole Normale Superieure de Lyon, FRANCE

## Abstract

Recent global declines, extirpations and extinctions of wildlife caused by newly emergent diseases highlight the need to improve our knowledge of common environmental factors that affect the strength of immune defense traits. To achieve this goal, we examined the influence of acidification and shading of the larval environment on amphibian skin-associated innate immune defense traits, pre and post-metamorphosis, across two populations of American Bullfrogs (*Rana catesbeiana*), a species known for its wide-ranging environmental tolerance and introduced global distribution. We assessed treatment effects on 1) skin-associated microbial communities and 2) post-metamorphic antimicrobial peptide (AMP) production and 3) AMP bioactivity against the fungal pathogen *Batrachochytrium dendrobatidis* (Bd). While habitat acidification did not affect survival, time to metamorphosis or juvenile mass, we found that a change in average pH from 7 to 6 caused a significant shift in the larval skin microbial community, an effect which disappeared after metamorphosis. Additionally, we found shifts in skin-associated microbial communities across life stages suggesting they are affected by the physiological or ecological changes associated with amphibian metamorphosis. Moreover, we found that post-metamorphic AMP production and bioactivity were significantly affected by the interactions between pH and shade treatments and interactive effects differed across populations. In contrast, there were no significant interactions between treatments on post-metamorphic microbial community structure suggesting that variation in AMPs did not affect microbial community structure within our study. Our findings indicate that commonly encountered variation in the larval environment (i.e. pond pH and degree of shading) can have both immediate and long-term effects on the amphibian innate immune defense traits. Our work suggests that the susceptibility of amphibians to emerging diseases could be related to variability in the larval environment and calls for research into the relative influence of potentially less benign anthropogenic environmental changes on innate immune defense traits.

## Introduction

Although it is well accepted that phenotypes vary between populations and are influenced by environmental conditions, there is increasing interest in the effects of environmental change on traits that affect resistance to newly emerging pathogens [1,2,3]. There is a large body of evidence indicating that environmental change, including human induced changes such as increasing temperatures, deforestation, and acidification, can alter an organism’s growth, development and survival [4,5, 6,7,8,9]. However, relatively few studies have experimentally examined the effects of environmental change on immune defense traits, which may greatly affect individual health [10,11,12, 13]. Given the rapid global spread of infectious diseases, a better understanding of the environmental factors that govern the expression of immune defense traits, and how this response to environmental change varies between populations and across life-stages, is increasingly needed.

Amphibians are an excellent study group for examining the role of the environment in regulating immune defense traits. Many of the diseases associated with amphibian declines either enter the amphibian through the dermal tissue (i.e. skin), or directly affect the dermal tissue (e.g. chytridiomycosis caused by the fungus *Batrachochytrium dendrobatidis*) [14,15,16]. Many amphibians possess two innate traits which resist pathogen infection of the skin. First, amphibian adults and larvae harbor diverse microbial communities on their skin [17]. Some amphibian skin-associated microbial species produce metabolites that suppress and eliminate some amphibian diseases [18,19,20,21,22,23]. Second, antimicrobial peptides (AMPs) produced by the granular glands of amphibian skin provide an effective defense against a variety of pathogens by disrupting pathogen cell and viral membranes [24,25,26]. How changes in the environment affect skin-associated microbial communities and AMPs has not been widely examined [27].

The small number of studies which have examined the effect of the environment on amphibian innate immune defense traits are largely correlative [17,28,29] and few have applied experimental manipulations to examine how environmental factors affect these traits [11,12,30,31,32]. Experimental studies provide conflicting evidence on the degree to which the environment may influence amphibian immune defense traits; some studies have found that immune defenses are not altered by the environment [11,12,17] whereas other studies have found environmental effects on immune defenses [30,32]. Even commonly encountered variations to amphibian habitat may alter immune defense traits as has been routinely found in studies examining traits associated with growth and development [33,34]. Additionally, commonly encountered variations in the environment may affect immune defense traits across life stages. Several studies have found alterations to larval habitat including changes in canopy cover, pond ephemerality, pollutants, predator exposure, and competitor densities can have long-term effects on amphibian growth, survival, and performance [33,35,36,37,38,39,40]; however, only two studies, that we are aware of, have examined carry-over effects of the environment on amphibian innate immune defense traits [11,31]. These two studies found significant effects of larval exposure to predators and competitors on post-metamorphic AMP production; however, the skin-associated microbial community was not examined. To improve our understanding of environmental influence on amphibian innate immune defense traits, additional studies are needed which 1) manipulate other commonly encountered amphibian environmental conditions 2) examine multiple immune defense traits in unison, and 3) assess the influence of the environment across life stages and populations. Knowledge of intraspecific differences in response to environmental change will improve our understanding of the relative importance of genetics and the environment on this aspect of amphibian health.

To test if commonly encountered variations in the environment simultaneously alter amphibian immune defense traits (i.e. skin-associated microbial communities and AMPs), we used the American Bullfrog, *Rana catesbeiana* (also known as *Lithobates catesbeianus*, sensu Frost et al [41]), as our model organism. We chose the American Bullfrog because of its high degree of environmental tolerance and introduced global distribution [42]. In our study, we hypothesized that commonly encountered variation in the larval habitat, small pH shifts (i.e. from ~ 7 to 6) and the presence or absence of pond shading (similar to canopy cover), can alter the microbial communities and AMPs of *R*. *catesbeiana* skin with little change to traditional correlates of amphibian fitness (survival, time to metamorphosis, and juvenile mass). Additionally we predicted that the treatment effects may differ between *R*. *catesbeiana* populations and that microbial community structure would change with ontogeny. Expecting that these common environmental variations may affect these innate immune defense traits in concert, we predicted that treatments affecting AMPs would similarly influence the post-metamorphic (juvenile) microbial community.

## Methods

### Experimental set-up

We conducted our experiment in 80 circular polyethylene tanks (1,100 liter), hereafter called “mesocosms”, located at Case Western Reserve University’s Squire Valleevue Farm (Hunting Valley, Ohio). On June 2, 2011 we filled each mesocosm with local pond water. Pond water was filtered using Phiefer Pet Screen to prevent predacious macro-invertebrates from being transferred into the mesocosms. We added approximately one gallon of dry leaves, collected from the mixed temperate hardwood forest floor adjacent to the mesocosm field, to each mesocosm to provide substrate for microbial growth and shelter for larvae. To prevent invasion by other species, tight-fitting screen lids made of 60% shade cloth covered each mesocosm.

We used a randomized block design with three treatments, each of which had two levels: population (larvae collected from two sites, one from southern Ohio and one from northern Ohio), acidification (acidified or un-manipulated pH), and canopy cover (shade or full sun) for a total of eight treatment combinations in five spatial blocks across the mesocosm field. We replicated each treatment ten times, for a total of 80 experimental units. To compare the effects of canopy cover, tent canopies (approximately 3 x 3meters) were randomly placed (by block) above half of the mesocosms on June 15, 2011. To acidify the larval habitat we manipulated water pH so that the acidified treatment had a pH of 5.5–6.5 (mean = 6.0 ±0.6), while the un-manipulated treatment had a pH of 7.0–7.5 (mean = 7.1 ± 0.5). To generate and maintain the lower pH we added hydrochloric acid (HCL) and lowered the pH by approximately 0.2 pH units per day beginning on 17 June. Acidification had three steps. First, 5 buckets each containing approximately 12 liters of water were acquired from each mesocosm. Second, we added 30% HCL to the buckets of water via micropipetter based on pH reading of the water (e.g. if 5ml of 30%HCL was to be added to the mesocosm, 1ml was administered to each bucket). Third, we slowly and gently poured each bucket of acidified water back into the mesocosm. Buckets were poured so as to thoroughly disperse the acid, mixing the solution into the entire mesocosm water volume, which prevented direct exposure of larvae to the concentrated acid product. To equalize disturbance across all mesocosms, we also removed and replaced the same volume of water in the un-manipulated treatments. We monitored pH on a daily basis using an Extech pH meter (model #PH100). “Acidified” mesocosms reached the desired level of difference from “un-manipulated” mesocosms ten days after experimental acidification initiation. Once pH equilibration was reached, acidification procedure was decreased to 1–2 times per week.

We collected 2000 hatchling *R*. *catesbeiana* larvae (Gosner stage 24–26) during the first two weeks of June 2011from each of two Ohio pond sites: southern Ohio (Butler Co.) and northern Ohio (Wood Co.). The sites differ dramatically in terms of anthropogenic influence. The southern Ohio site receives water from treated domestic waste-water effluent, is largely unshaded with little canopy cover, and is presumed to experience higher levels of pH instability due to runoff from the adjacent golf course and the chemicals used by pond owners to control pond algal blooms (e.g. copper sulfate). We measured pH at the southern Ohio site on June 6, 2011 and found it was 9.74 (ExTech model #PH100). In contrast, the rural northern Ohio pond site is a protected pond (Wood County Park District) that is partially shaded from the sun with forest surrounding the pond’s north, east, and west regions of the pool, with long-term fallow fields of prairie plants and hawthorn trees at the pond’s southern side. We measured pH at the northern Ohio site on June 11, 2011 and found it was 8.95 (ExTech model #PH100). The southern Ohio population is located approximately 220km south of the northern Ohio population’s collection site. Site access was permitted by landowners.

We added 50 *R*. *catesbeiana* larvae to each of the 80 mesocosms. Southern Ohio larvae were collected on June 6, transported on June 7, and added to mesocosms on June 8, 2011 (a random sample of 10 tadpoles were all Gosner 25). Northern Ohio larvae were collected on June 11 and 12, transported on June 12 and added to mesocosms on June 13, 2011 (average Gosner Stage: 25.1 +/- 0.11, N = 10) [43]. Larval diet was supplemented with rabbit chow (Kaytee) throughout the duration of the larval period to maintain adequate food availability for all larvae. Supplemental food was administered equally across all mesocosms 2x weekly (3.5g/mesocosm).

When larvae reached Gosner Stage 42 (i.e. when front legs erupt), we transferred the first three metamorphosed juvenile frogs from each mesocosm to an indoor animal room maintained at 28° C with a 12 hour light/dark cycle. While *R*. *catesbeiana* commonly overwinter as larvae and may take up to three years to reach metamorphosis [44], high temperatures, low densities, and associated higher food availability can facilitate rapid growth and development [45,46,47]. *Rana catesbeiana* in the Midwestern United States are known to reach metamorphosis within a single season [45]. We did not manipulate pH and shade in post-metamorphic habitats. After three juvenile *R*. *catesbeiana* individuals had been transferred from each mesocosm to the indoor laboratory facility, the remaining larvae in that mesocosm were collected and counted. Three larvae were swabbed for microbial community analyses (see sample collection description below) to examine treatment effects on the skin-associated microbial communities of larvae. Larvae were subsequently euthanized using MS-222. Unfortunately, across the 4000 larvae that were introduced to the mesocosms, eleven were determined to be another species (*Acris crepitans*); however all survival data (percent survival) was corrected for this error. At the indoor laboratory facility, juvenile *R*. *catesbeiana* were housed in 15L polyethylene boxes held at a slant (~15 degrees) so that the 1L of de-chlorinated water in each box was deeper at one end providing both terrestrial and aquatic regions. Plastic cups provided shelter. Juvenile *R*. *catesbeiana* were fed five crickets per animal three times per week and water was changed 3x weekly (100% water change).

### Data collection and analysis

Percent larval survival was determined by counts of larvae remaining at the end of the larval rearing period and was log transformed. Due to unexplained, extremely high mortality in a single mesocosm (only one animal reached the end of the experiment), this mesocosm was eliminated from all analyses. Average survival in all other mesocosms was 95.3% ± 1.3% SE. Average time to metamorphosis per mesocosm was found by determining the average number of days from experiment beginning (date of larval addition) until date of metamorphosis for the three juvenile frogs transferred to the indoor laboratory facility. We log transformed average time to metamorphosis to meet normality. We assessed treatment effects on percent survival and average time to metamorphosis utilizing ANOVA (Type III sums of squares). Each response variable was regressed on to all treatments (Acidification, Population, Shade), interactions between treatments, and block. Mass (g) was obtained for each juvenile frog immediately post euthanasia (post microbial community sampling and AMP collections) and was averaged by mesocosm. Mass was cubed to meet normality [48]. Treatment effects on juvenile mass were assessed with ANCOVA to account for possible confounding effects of when (age in days) mass data was collected in respect to date of metamorphosis (predictor variable called “Days in lab” hereafter). All three post-metamorphic animals from a single mesocosm died during the laboratory rearing portion of the study, and subsequently, this mesocosm was excluded from all post-metamorphic trait analyses (juvenile survival was 100% after excluding this mesocosm).

We collected microbial community samples of larvae and juvenile frogs using sterile swabs (product # MW113, Advantage Bundling), pre-rinsing animals in sterile water and subsequently gently rubbing the swab across the animal’s skin in a standardized manner [17]. Microbial samples taken from juvenile frogs were collected immediately prior to AMP collection. Swabs containing skin microbial community samples were subsequently frozen at -80° C in 2ml cryovials until processed.

To avoid pseudo-replication, we pooled swabs by mesocosm and developmental stage (i.e. swabs from animals contained in the same mesocosm were analyzed as a single unit and larval swabs were analyzed separately from juvenile swabs). We extracted microbial DNA from the skin swabs using a bead beating and phenol chloroform extraction method [49,50]. Negative PCR results using two different primer sets (58A2F and NLB4, 58A2Fand ITS4) targeting the ITS2 gene region of fungal DNA suggested that fungal communities did not contribute significantly to the microbial community on the skin of the animals used in this study; therefore further fungal community analyses were not performed. If fungal communities had significantly contributed to the skin microbiome of animals in this study at either stage of development (larvae or juvenile), quantification of Bd, *Batrochochytrium dendrobatidis*, would have been conducted as a likely contributor to the fungal microbial community. We amplified bacterial DNA using 16S rRNA gene primers: 338f and 926r [51] according to the Burke et al. [50] protocol.

Using terminal restriction fragment length polymorphism profiling (TRFLP), we examined microbial community structure across treatments [49,50,52,53]. This profiling procedure provides results comparable to 454 pryosequencing when sampling across local spatial scales such as in this study [54]. We used restriction enzymes MspI and HaeIII (Promega) to prepare samples for TRFLP profile analyses subsequently generated at the Life Sciences Core Laboratory Center (Cornell University) using a GS600 LIZ size standard (Applied Biosystems). We used Peak Scanner Software (version 1.0, Applied Biosystems 2006) for our analyses. Only peaks which accounted for >1% of the relative peak area were included in sample analyses [53]. Only TRFs produced by MspI restriction enzyme with the reverse primer were included in analyses because HaeIII digests did not produce adequate fragment numbers. We used nonmetric multi-dimensional scaling analyses (NMDS) and multi-response permutation procedures (MRPP) to assess treatment effects on bacterial community structure in PC-ORD (Version 5.0; Bruce McCune and MJM Software, 1999) for larvae and for juvenile frogs. MRPP is a non-parametric discriminant function analysis which tests for difference between two or more groups of entities. TRFLP profiles were arcsine-square root transformed prior to analysis [55]. We utilized a cloning and sequencing approach to identify dominant members of the larval and juvenile frog skin-associated microbial community (Qiagen PCR Cloning Plus) constructing two clone libraries (Larvae N = 78, Juveniles N = 83) for larval and juvenile frog bacterial communities. We archived resulting cloned sequences in GenBank (Appendix A; Accessions HF947349-HF947509). Indicator species analyses were conducted on terminal restriction fragments which were identified to taxa using predicted TRFs from the clone libraries. Indicator species analysis (a monte carlo test) was completed using PC-ORD (version 5.0) and determines whether bacterial species on *R*. *catesbeiana* skin differed between treatments or life stages.

We collected AMP samples from juvenile frogs on September 15–17, 2011 using a modified protocol by Rollins-Smith [56] utilizing a 0.01% nor-epinephrine bath to elicit the secretion of AMPs by juvenile frogs [57]. AMP samples were collected grouping frogs by mesocosm to avoid pseudo-replication. Each group of frogs was placed in the nor-epinephrine bath (500µl of 20mM nor-epinephrine hydrochloride in 50ml of collection buffer; collection buffer consists of 2.92g NaCl, 2.05g sodium acetate and 1L of HPLC grade water). The bath covered the frogs’ bodies. Collection vessels were swirled to wash proteins from the frogs' skin and to prevent frogs from climbing out of the bath. After 15 minutes the solution was removed from the collection vial. The collected buffer (and secretions contained within) was then immediately acidified with 100% TFA and filtered using a C-18 Sep-Pak Classic Cartridge (Waters Corporation) and Sep-Paks were subsequently rinsed with 1%TFA before storing. All juvenile frogs had completely absorbed tails prior to sample collection. Samples (C-18 Sep-Paks) were frozen at -80° C until sample elution in parafilm sealed falcon tubes to prevent desiccation. Eluted samples were dried at 15° C in an Eppendorf Vacufuge^.^ Samples were reconstituted in 1ml of sterile water (HPLC grade) and syringe filtered (13mm Pall Acrodisc with Tuffrynmembrane and 0.2µm pore size). We utilized a Micro BCA Protein Assay Kit (product # 23235) for analysis of total protein concentration from our AMP sampling. We used 100µl reactions to measure optical density at 562nm (absorbance) with a BioTek Synergy HT plate reader. Absorbance measures were used to estimate concentration of the protein (µg/ml) using Bradykinen as the protein standard (i.e. AMP production). Each sample and standard was run in triplicate. The concentrations of the protein standard were log transformed and a linear model was used to estimate protein concentration within each sample. AMP production was averaged by mesocosm and standardized by total frog mass (i.e. mass of the three juvenile frogs sampled was summed and µg/ml AMP was divided by this total mass) and log transformed to meet normality. We standardized the measure of AMP production by frog mass because larger frogs have more skin and therefore are likely to produce more secretions. Standardizing by frog mass allows for cross treatment comparisons without the potential confounding effects of the size of the frogs on this measure of AMP production. We analyzed AMP production with ANCOVA (Type III) by regressing AMP production (µg/ml per gram body weight) onto all predictor variables (Acidification, Population, Shade), block and AMP collection time in respect to date of metamorphosis (number of “Days in lab” before AMP sampling), including interactions between Acidification, Population and Shade. Heteroscedasticity of the model was quantitatively assessed via a Breusch-Pagan test, and the assumption of homogenous variances was confirmed.

We conducted assays against *Batrochocytrium dendrobatidis* (Bd strain JEL 404, originally isolated from a *R*. *catesbieana* larva in Oxford Co. Maine) in culture to determine bioactivity of AMP samples. Based upon the BCA assay results, standardized concentrations of each AMP sample were made. Final concentrations of 40µg/ml, 20µg/ml, 10µg/ml, 5µg/ml, and 1µg/ml were tested against Bd using a microplate technique. 50µl of Bd zoospore solution at a concentration of 2 x 10^6^ zoospores/ml (in 1% tryptone broth) was added to each well of a 96 well flat-bottom sterile plate. 50µl of AMPs at the aforementioned concentrations was then added to each well (each concentration for each sample replicated 3 times). We prepared positive and negative controls on each 96 well plate (three replicates per control on each plate). Positive controls consisted of 50ul of 2 x 10^6^ Bd zoospores and 50ul of sterile PCR grade water and negative controls contained 50µl of heat killed Bd zoospores of the same concentration and 50µl of sterile PCR grade water [58,59]. We read optical density (OD; BioTek Synergy HT) of wells at 490nm on days 0 (immediately after plating), day 1(13 hours post plating), day 2, day 3, day 5, day 7, day 9, and day 11. Zoospore growth of all samples had plateaued by day 9. Percent growth was determined for each sample (mesocosm) by subtracting mean OD490nm on day 9 from mean OD490nm on day 1 and multiplying by 100 for each sample. Bioactivity was defined as the slope of the best fit line calculated from the log transformed growth curve for each sample [59]. We could not determine minimal inhibitory concentration (MIC) in our bioassay because it was greater than 40 µg/ml; for this reason, our log transformed growth curves are linear, allowing for bioactivity to be assessed using the slope of the log transformed growth curves as the response variable in our models. We suspect our inability to assess the MIC is because recently metamorphosed juvenile bullfrogs produce relatively few AMPs. It is unknown at what point in post-metamorphic development that amphibians are capable of producing their full repertoire of AMPs [60]. We analyzed bioactivity (slope) with ANCOVA (Type III) by regressing slope onto all predictor variables (Acidification, Population, Shade), block, and Days in lab, including interactions between Acidification, Population and Shade.

Due to the fact that not all bioassay samples show plateaued growth (OD490) on the same day (range Day 3-Day 9), we examined potential treatment effects on a second measure of bioactivity, growth rate. A logistic growth model was fit to data using a self-starting nls logistic model function (R Development Core version 3.0.2, stats package, José Pinheiro and Douglas Bates) for all samples at a concentration of 20µg/ml using a reparameterized version of the logistic growth model (Formula A: below), where “P” is the population size, “Po” is the original population size (population sizes measured as OD490nm), “t” is time in days, “K” is the carrying capacity (plateau point of Bd growth), and “r” is the growth rate.

P=K/1+ePo+rt Equation 1

Twenty µg/ml was the highest peptide concentration in which all samples were represented. Growth rate “r” was then assigned as the response variable and regressed onto all predictor variables (Acidification, Population, Shade), block and Days in lab, including interactions between Acidification, Population and Shade in an ANCOVA (Type III) model.

Unless otherwise stated, we completed statistical analyses using R [61]. All ANOVA and ANCOVA models were assessed using referent cell coding (*treatment contrasts* as opposed to *helmert contrasts*; [62]) examining the effects of each treatment combination on each response variable as a separate model. This methodology provides assessment of treatment effects within three-way interaction models by conducting ANOVA/ANCOVA for each treatment combination independently, comparing within-group means [63]. Results are described using p^range^ indicating a range of p values for each response across treatments.

This study was carried out in strict accordance with guidelines of the Ohio Department of Natural Resources (permit number 14–222) and approved by Case Western Reserve University’s Institutional Animal Care and Use Committee (IACUC permit number 2011–0073).

## Results

**Fig 1:**
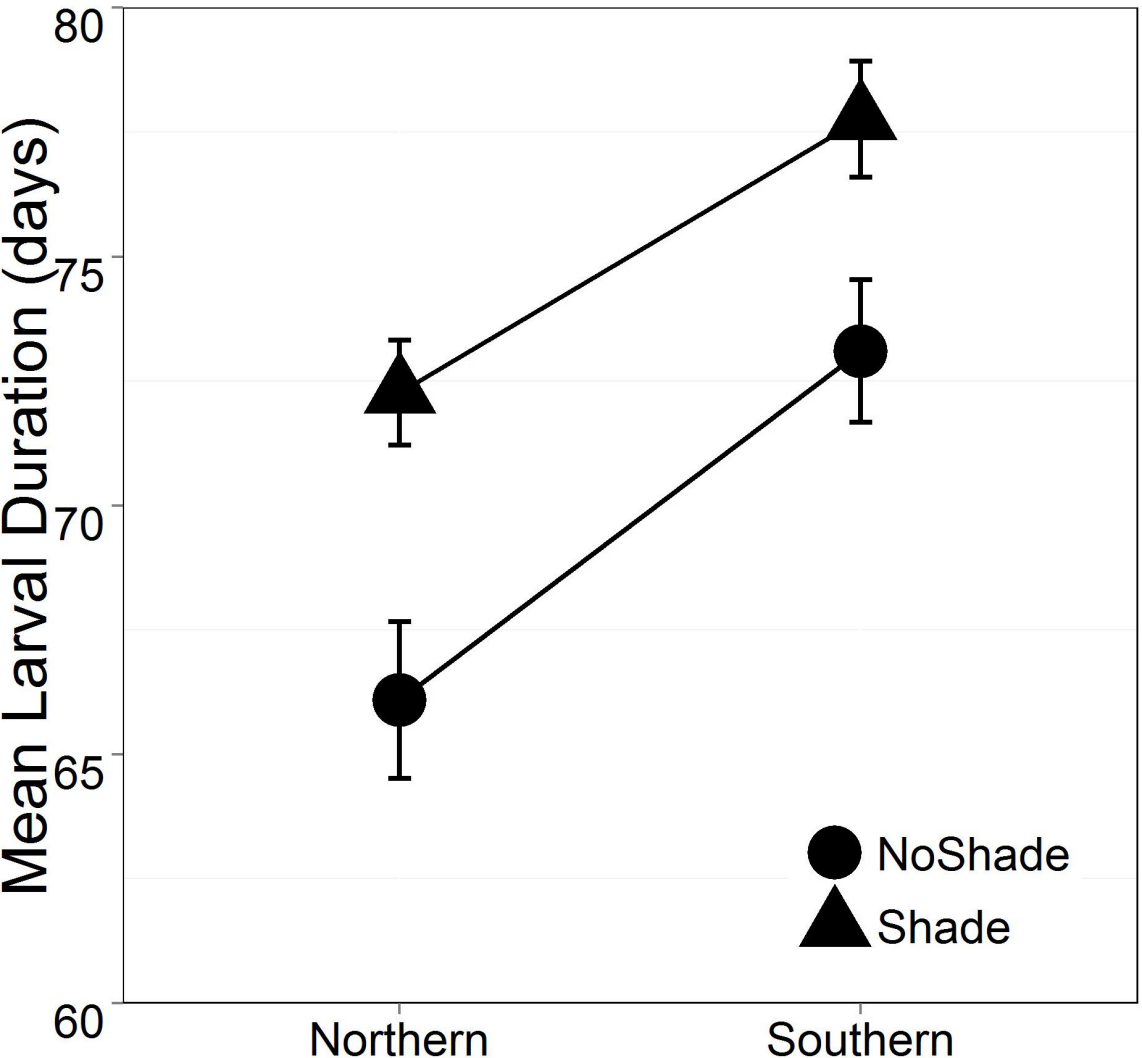
Average time to metamorphosis with standard error. Both Shade and Population were significant predictors of mean larval duration under all treatment combinations (shade p^range^ = 0.003 to 0.04; population p = 9.5 x10^-5^–0.017). Figure displays results of Population effects within Acidified environments. Full ANOVA outputs can be found in S1 Table.

**Fig 2:**
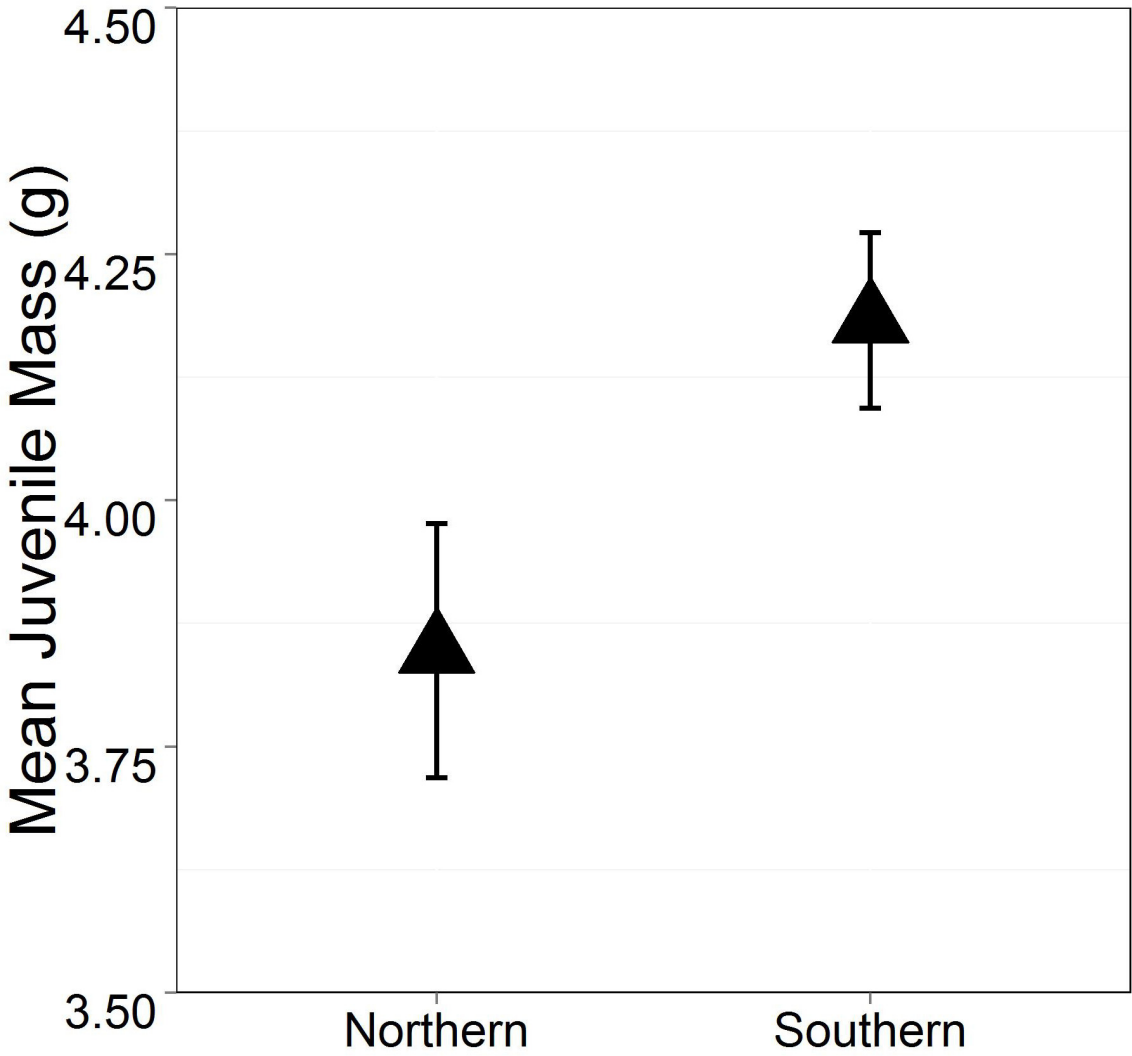
Population effect on mean juvenile mass (g) at sample collection with standard error. Population and Days in lab were significant predictors of juvenile mass in many but not all treatment environments (Population p^range^ = 0.007 to 0.0866; Days in lab p = 0.0042). Figure displays results of Population effects within Shaded and Acidified treatments. Full ANCOVA outputs can be found in S2 Table.

While we found no significant treatment effects on larval survival (Mean: 95.3% ± 1.3% SE), there were treatment effects on the other larval traits. Shade significantly delayed average time to metamorphosis (mean larval duration: shaded mesocosms 75.12 ± 0.56 days, unshaded mesocosms 69.35 ± 0.83 days SE; p^range^ = 0.0033 to 0.0395; Fig 1, S1 Table); however Acidification did not have a significant effect on average time to metamorphosis (p^range^ = 0.4767 to 0.9766). The southern population had significantly longer larval duration than the northern population (mean larval duration: southern Ohio 75.92± 0.75 days, northern Ohio 68.38 ±0.55 days; p^range^ = 9.5 x 10–5 to 0.0165; Fig 1, S1 Table). Population also significantly affected juvenile mass (Mean: southern Ohio 4.28 ± 0.04g, northern Ohio 3.90 ± 0.06g, p^range^ = 0.0072 to 0.0866; Fig 2, S2 Table), even when taking duration of time between metamorphosis and sample collection into account (Days in lab p = 0.0042). In other words, juvenile frogs held in the indoor laboratory facility for a longer period of time were greater in mass. Acidification and Shade treatments did not significantly affect juvenile mass at sample collection. No interactions were significant for any of these models.

**Fig 3:**
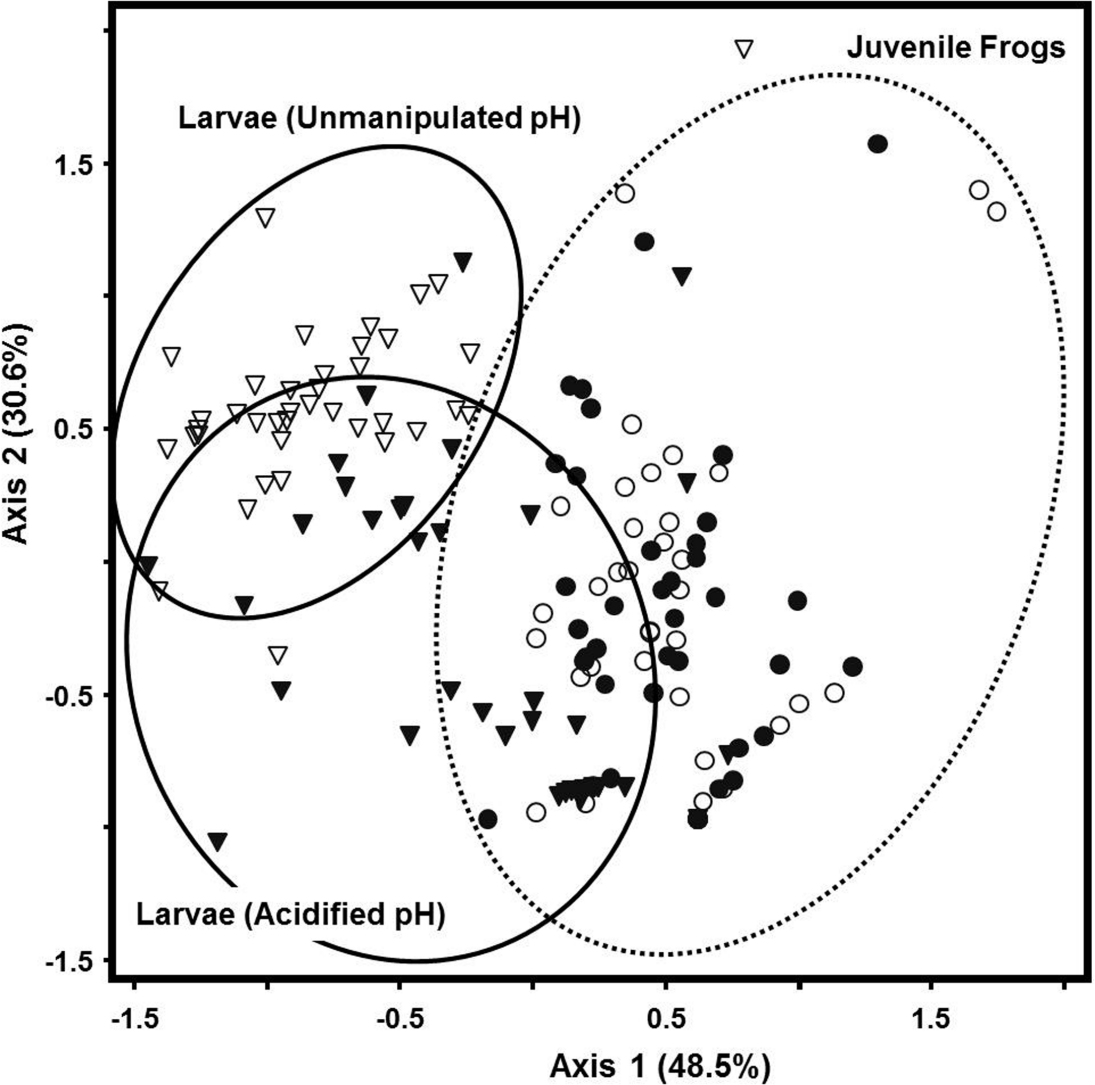
NMDS ordination plot of *R*. *catesbeiana* larval and juvenile frog microbial community similarity by acidification treatment. N = 152 after outlier analysis (McCune and Grace 2002). Ordination stress = 20. Axes display percentage variance explained. Circles designate juvenile frog microbial communities, triangles designate larval microbial communities. Open symbols designate acidified pH treatments while closed symbols designate un-manipulated pH treatments.

**Fig 4:**
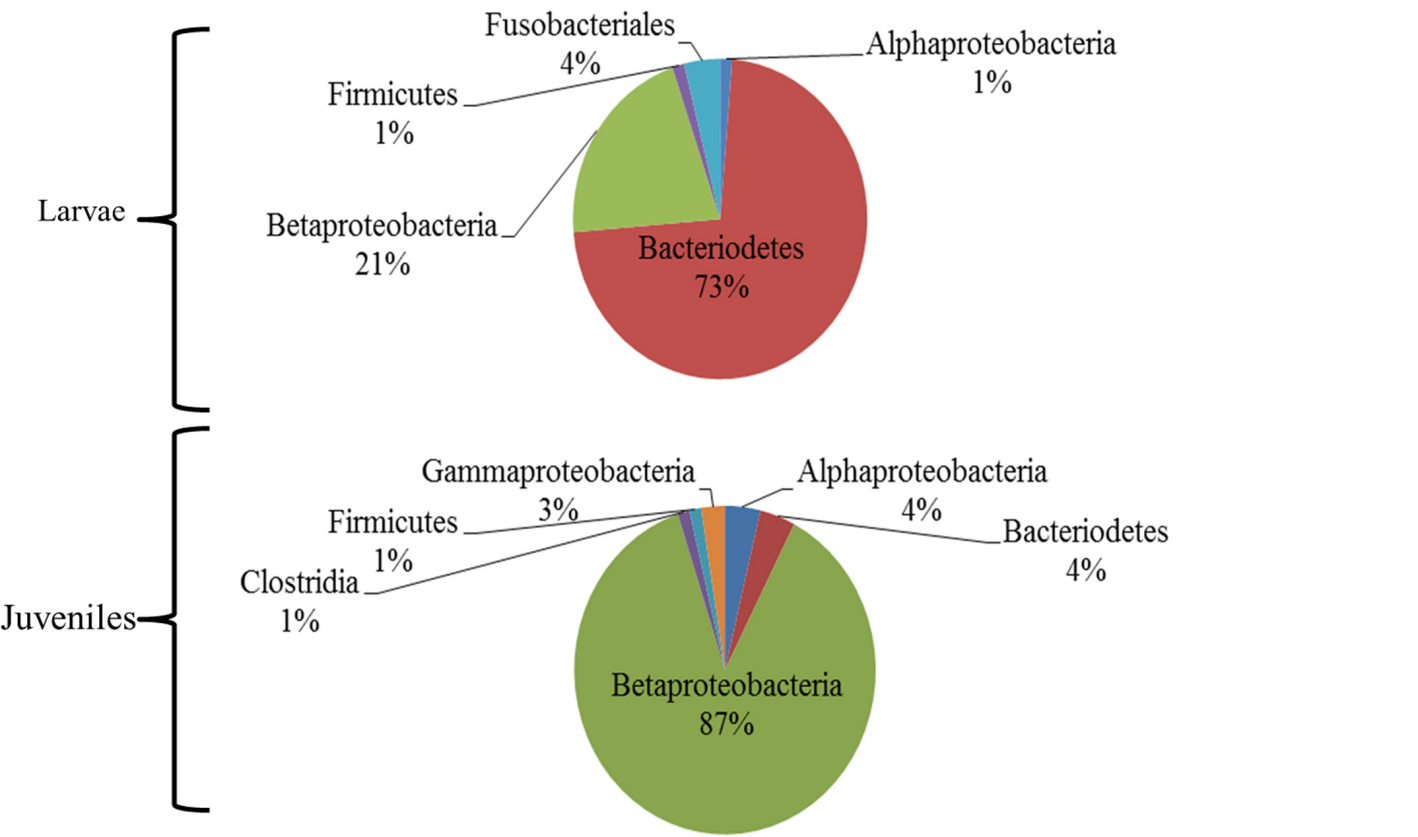
Clone library comparison between larval and post-metamorphic (juvenile) *R*. *catesbeiana* skin-associated bacteria. The percent of the clone library represented by each taxonomic group is shown. (Larvae library: N = 78, Juvenile library: N = 83)

NMDS and MRPP analyses indicated differences in microbial community structure between developmental stages (larvae and juvenile frogs) (A = 0.10, p<0.0001, Table 1, Fig 3). Within the larval stage, acidification of the larval habitat altered skin microbial communities (A = 0.14, p<0.0001, Table 1, Fig 3). Our examination of juvenile frog microbial community structure did not reveal any significant treatment affects (Table 1). Clone library comparisons highlight the large difference in skin-associated microbiota between larvae and juveniles most notably in terms of a shift from a Bacteriodetes dominated (73%) larval flora to a Betaproteobacteria dominated (83%) juvenile frog flora (Fig 4, S6 Table). Multiple indicator species of developmental stage (using predicted terminal restriction fragment size) were also found including the genus *Herbaspirillum* which is only represented in the juvenile frog clone library and *Cetobacterium* only represented in the larval clone library. *Ideonella sp*. was an indicator of acidified treatment while *Niastella sp*. was an indicator of non-acidified treatment within the larval clone library.

**Table 1:** MRPP results from microbial community comparisons.

Grouping Factor	Treatment	A	p
Combined samples (larvae and metamorphs)	Developmental Stage	**0.1**	**<0.0001**
	Acidification	0.04	<0.0001
	Shade	0.004	0.0588
	Population	0.0007	0.2819
	Block	0.004	0.1651
Larvae	Acidification	**0.140**	**<0.0001**
	Shade	0.0129	0.0365
	Population	0.001	0.3039
	Block	0.0021	0.3658
Juvenile Frogs	Acidification	-0.0056	0.9596
	Shade	-0.0029	0.7071
	Population	0.0006	0.3688
	Block	0.0276	0.0062
	Acidification x Shade	-0.0077	0.8442
	Population x Shade	-0.0084	0.872
	Population x Acidification	-0.0088	0.8866

Significance (bold) defined as an Affect Size (A) where A≥0.1and p≤0.05 (McCune and Grace 2002).

**Fig 5:**
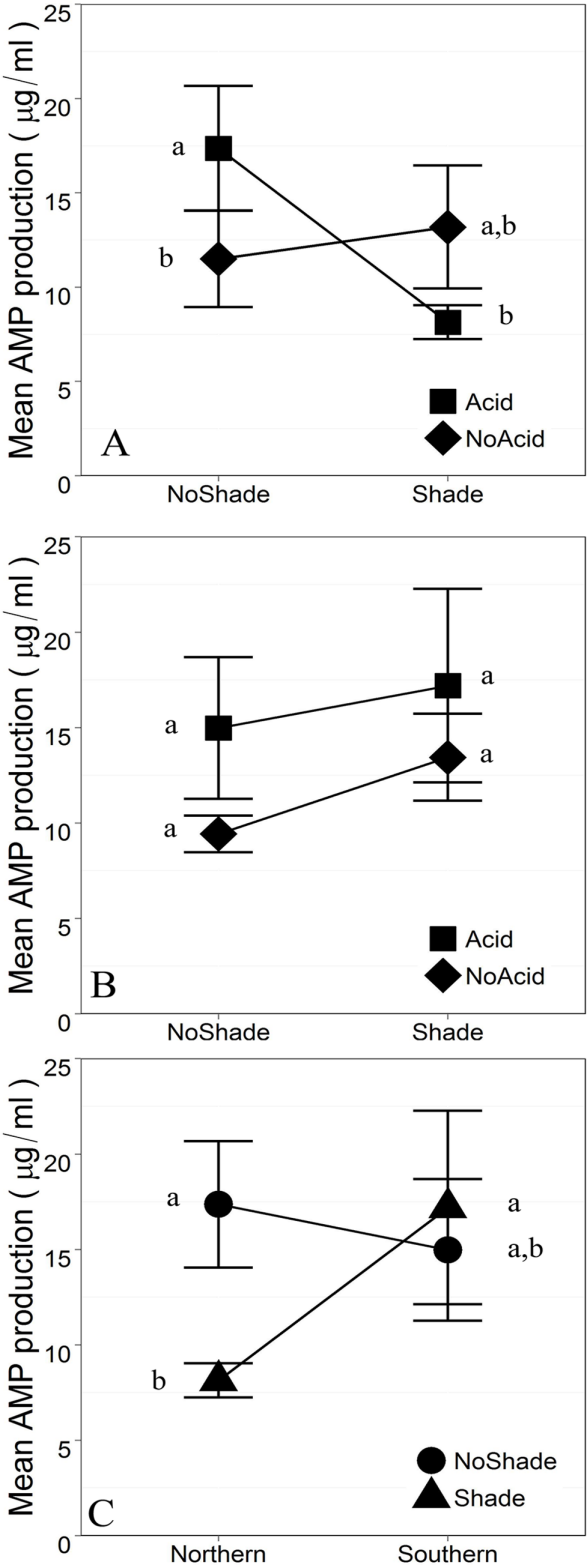
Interaction effects on AMP production (µg/ml standardized by gram body weight) with standard error (Acidification x Shade p = 0.0272; Population x Shade p^range^ = 0.0501–0.7868). A. Northern referent. B. Southern referent. C. Acidified referent. Referent variables refer to a specific treatment environment, indicating what two-way interaction is being displayed. Contrasts indicate significant simple effects within each two-way interaction (p<0.05) (eg. A. indicates a significant Acidification effect within the NoShade treatments and a significant Shade effect within the Acidified treatments in the northern Population) [62,63]. Full ANCOVA outputs can be found in S3 Table.

Antimicrobial peptide (AMP) production analyses revealed significant Acidification X Shade (p = 0.0272) and Population x Shade (p = 0.0501) interactions across many, but not all treatment combinations (Fig 5; S3 Table). These results indicate that the populations utilized in our study responded differently to larval habitat acidification and shading.

**Fig 6:**
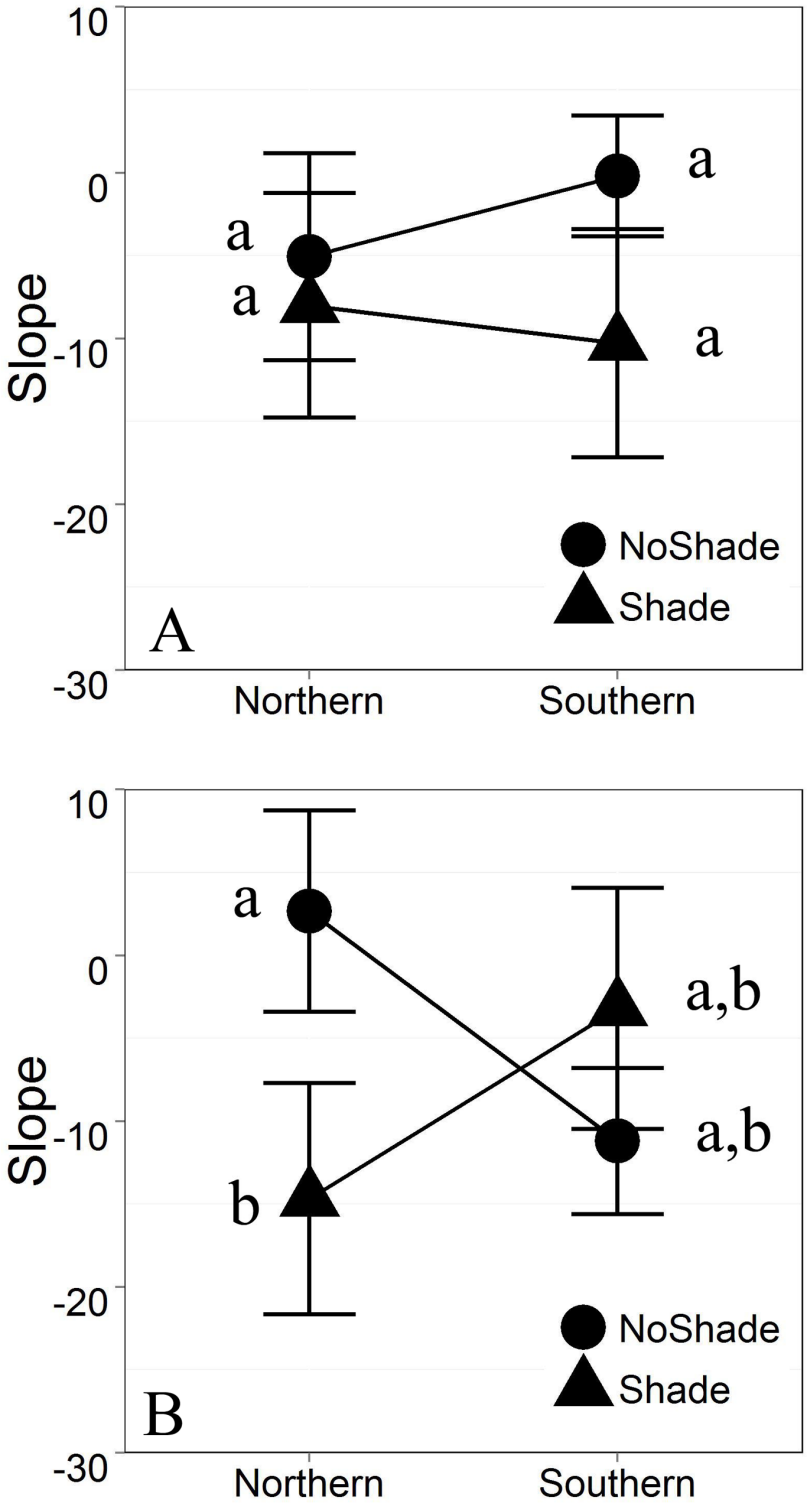
Interactive effects on AMP bioactivity in terms of slope of the log-transformed growth curve with standard error (Shade x Population p = 0.085, Acidification x Shade x Population p = 0.12). A. Acidified referent. B. No Acid referent. Contrast indicates significant simple effect of Shade within un-manipulated pH (NoAcid) treatments of the Northern population (p = 0.018). Full ANCOVA results can be found in S4 Table.

**Fig 7:**
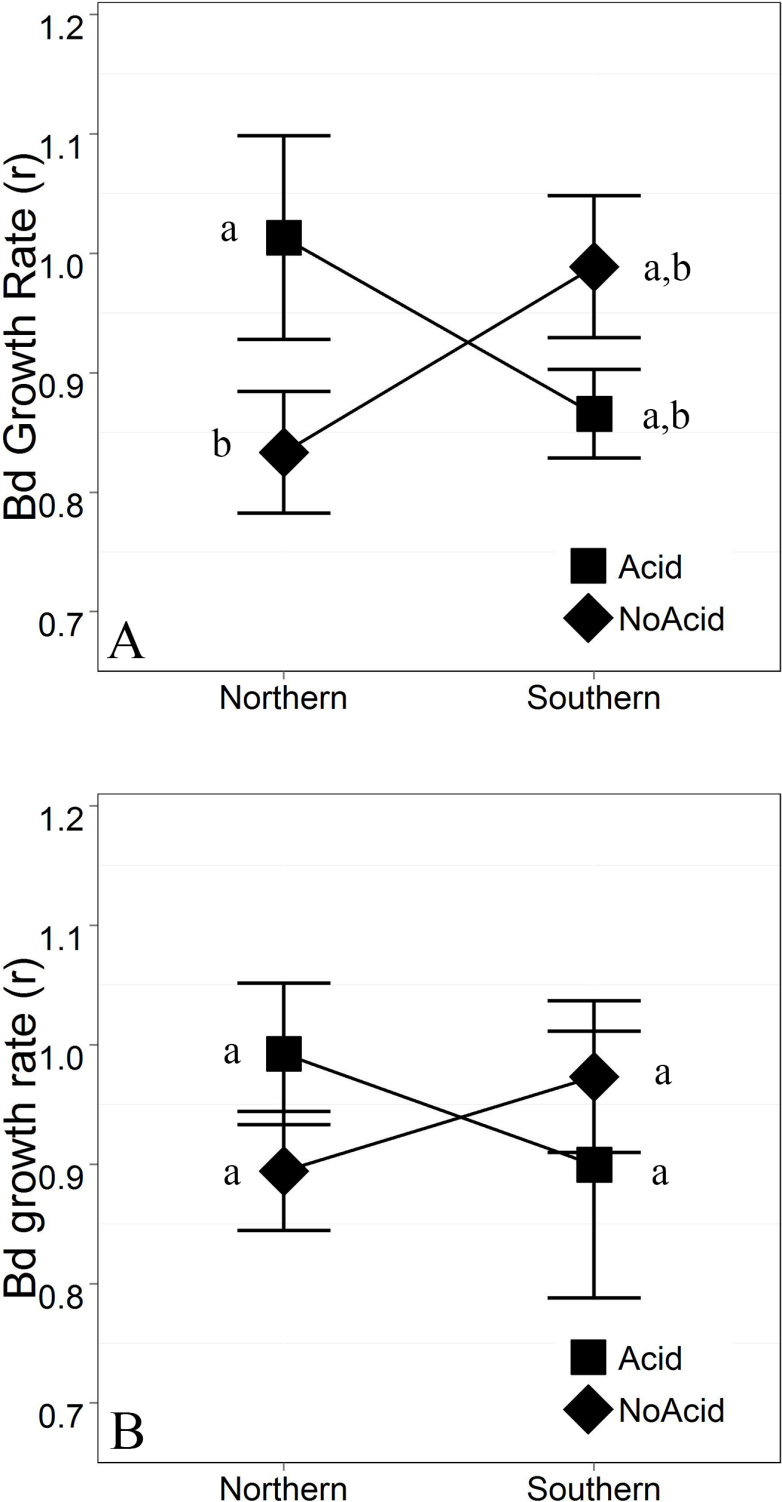
Interactive effects on AMP bioactivity in terms of Bd growth rate with standard error (Acid x Population p^range^ = 0.033–0.084, Acidification x Shade x Population p = 0.773). A. Sun referent. B. Shade referent. Contrast indicates significant simple effect of Acidification within full sun (NoShade) treatments of the Northern Population (p = 0.018). Full ANCOVA results can be found in S5 Table.

Antimicrobial peptide (AMP) bioactivity analyzed as slope of the log transformed growth curves showed significant main effects of Shade (p = 0.0175) and marginal Population x Shade interaction effects (p = 0.085) in some but not all environments; again, indicating that the populations utilized in this study are responding differently in terms of AMP bioactivity (slope), though our detection of a three-way interaction was marginal (p = 0.118; Fig 6, S4 Table). When bioactivity was assessed using Bd growth rate “r” calculated from the logistic growth model, we found significant (or marginally significant) Population X Acidification interactions (p^range^ = 0.0327–0.0839; Fig 7, S5 Table). This final measure of bioactivity in terms of Bd growth rate indicates population level differences in response to larval habitat pH change.

## Discussion

Recent disease-associated declines, extirpations, and extinctions of amphibians world-wide have resulted in numerous studies which examine relationships between disease resistance and innate immune defense traits [19,26], but little is known about the influence of the environment on these traits, or how consistent responses to environmental variations may be across populations [20,27]. Our findings support the hypothesis that common variation in the larval environment can significantly alter amphibian immune defense traits. By measuring both skin-associated microbial communities and antimicrobial peptides we gain additional information to assess amphibian fitness beyond the commonly measured correlates of fitness, traits such as survival, time to metamorphosis and juvenile mass. While larval duration and juvenile mass were affected by pond shading and population, these traits were not affected by larval habitat acidification. Larval survival was not affected by any of our treatments. Microbial community structure was affected by our small changes to larval habitat pH (i.e 1 pH unit), but this effect of pH did not carry-over post-metamorphosis. We did not find effects of pond shading or population on microbial community structure in either larvae or juvenile animals. Post-metamorphic AMP production and bioactivity however revealed complex interactions between these larval habitat changes and population in addition to indicating that the larval environment has a legacy effect on AMPs expressed after metamorphosis.

We found that a pH change of 1 unit, near neutral, did not alter the commonly measured correlates of fitness (e.g. survival, time to metamorphosis, juvenile mass). The effects of pH changes near neutral (pH 7) have not been shown to affect survival, but can cause changes in larval growth [64,65]. However, low pH (≤4.7) has been shown to negatively affect survival, larval duration, juvenile mass, and can indirectly alter these traits through interspecific interactions [66,67,68,69]. In our study, an average change from pH of 7 to pH of 6 in the larval habitat yielded surprising strong effects on the microbial community inhabiting the skin of larval *R*. *catesbeiana*. The mechanism by which these composition shifts occur is unknown. It is also unknown if this change in the microbial community results in functional differences and if this change in microbial community affects the larvae’s ability to resist disease. However, if skin-associated microbial communities are an important defense against pathogens, it is conceivable that the changes we observed could influence disease resistance. Meta-transcriptomic approaches may assist future studies in assessing functional differences between skin-associated microbial communities that develop from changes in pond water pH [12]. Bacteria isolated from amphibian skin can produce metabolites that inhibit pathogens [21] and previous studies have noted that multiple bacterial species from the Class Betaproteobacteria and Phylum Bacteriodetes, the dominate taxa present in our samples, can provide amphibians with pathogen resistance [23,70]. Microbial species could also contribute to immune defense by providing a physical barrier to infection or by stimulating the amphibians’ production of antimicrobial peptides (AMPs) which constitutes the second innate immune defense trait; therefore, environments which alter microbial community structure may also alter resistance to pathogens through AMP production. Conversely, environments which alter AMP production or relative proportions of AMP constituents may alter the microbial associations of the amphibian skin. While no studies have examined this in amphibians, similar relationships have been previously documented in human studies of skin-associated microbial communities and AMP production [71]. Microbial communities may also provide other benefits beyond disease resistance. For example, as has been documented with plants, microbial communities could be assisting their host organisms in processes such as osmoregulation and nutrient uptake [72,73]; therefore knowledge of how common variations in the environment alter these communities may be important for understanding amphibian health in ways that have yet to be explored.

Unlike the microbial community shift observed in our larval samples, pH of the larval environment did not have a significant effect on the microbial community structure of the juvenile frog skin. In other words, there was no evidence of carry-over effects of the larval habitat pH on the juvenile frog skin-associated microbial community. Our study did find significant shifts in the microbial community between larvae and newly post-metamorphic juvenile frogs. These results are similar to those found by Kueneman et al [28] which is the only other published study examining ontogenetic effects on the amphibian skin-associated microbiome. In that field-based study, microbial community structure differed between larvae and juvenile *Rana cascadae*, within a single site. The difference in skin-associated microbial communities between larval and post-metamorphic amphibians may be due to physiological changes undergone during metamorphosis or are associated with the more terrestrial behavior of the post-metamorphic frogs. It has been hypothesized that AMPs produced after metamorphosis may regulate microbial community structure [26,30]. If microbial community structure is regulated by the AMPs after metamorphosis, we would expect to see both AMPs and microbial community structure affected in similar ways by our treatments. However, our treatments did not affect post-metamorphic microbial community structure, suggesting that we can reject the hypothesized link between microbial community structure and AMPs in this case. It is important to consider that if AMP production was affected to a much greater extent, it is possible that this may shift the skin-associated microbial community.

Multiple hypotheses could explain the differences between populations in AMP production and bioactivity in response to our experimental treatments including differential ability of populations to plastically respond to our environmental perturbations, differences in maternal investment between populations, carry-over effects from early life-experiences prior to larval collection, or local adaptation [74,75,76,77]. We found significant increases in AMPs produced by animals from the northern population, which is in stark contrast to the lack of response by the southern Ohio population to our treatments. We suspect the southern Ohio collection site to be highly variable in terms of water quality as it is receiving water for treated residential sewage and is located next to a chemically treated golf course. Our mesocosm environments would therefore be more different from the native environment for the northern Ohio population (little natural variation in water quality) than the southern Ohio population (high variability in water quality). Consistent with the hypothesis of local adaptation or carry-over effects of early life experience, increased AMP production by the northern population may indicate a stress response caused by the relatively large change in environmental conditions in respect to the stable conditions the population has adapted to [26]. On the other hand, the lack of response by the southern population may reflect adaptation to highly variable and potentially stressful water quality conditions stemming from chemical contamination of the pond by human activities. Future studies may need to measure levels of corticosteroid or other stress associated hormones to elucidate potential mechanistic relationships between environmental change and stress response in terms of AMP production. AMP bioactivity of these natural peptide mixtures may also be decreased with increasing AMP production because of changes in the relative proportion of AMPs produced [26,59]. Future research should examine effects of such commonly encountered variations in the environment on AMP constituents as could be measured by high pressure liquid chromatography (HPLC) analyses [78]. This would allow us to examine how commonly encountered variations in the environment alter relative proportions of AMPs produced by different populations.

Our finding that common larval habitat changes carried-over to alter post-metamorphic AMP bioactivity was surprising and supports the hypothesis that the larval environment can have long-term effects on amphibian health. Few studies have examined the potential carry-over effects of the larval habitat on post-metamorphic immune defense traits [11,31]. While our two measures of AMP bioactivity provide somewhat conflicting results, this may be explained by a lack of statistical power to detect the three-way interaction between acidification, shade and population. This finding provides future researchers with rational for careful consideration of the likely complicated interactive effects on amphibian immune defense traits.

## Conclusions

We found that commonly encountered variation in environmental conditions can alter amphibian innate immune defense traits differentially across populations and life-stages. Natural environmental variation in soil chemistry (e.g. pH, alkalinity) is expected at a landscape level, due to changes in geology, climate or land cover. If immune defense traits, as found in this study, are affected by these natural changes, our results have implications for our understanding of differences in the magnitude of disease outbreaks and mortality between populations at the landscape level. Our research also has implication for our understanding of how anthropogenic change may differentially affect population immune defense traits and response to disease pressure. Global climate change, agrochemical usage and run-off, and invasive species interactions with native wildlife all have the potential to alter immune defense traits either directly or indirectly and quite possibly to a greater degree than our treatments induced, but studies of the effects of anthropogenic influence on immune defense traits and correlated responses of populations to disease pressure are currently lacking. In addition, our work suggests that future studies should incorporate multiple developmental stages in such analyses, for as we have shown, changes to larval habitat may have long-term effects on traits not measureable until later developmental stages. Many previous studies have shown species level differences in skin-associated microbial communities and AMPs [17,24,28,29] but population level variation of these traits and the influence of the environment on these traits across populations is an area of research which needs further exploration [27]. Such research programs have the potential to identify unforeseen direct and indirect effects of anthropogenic environmental changes to species’ immune defense traits and disease resistance capabilities, providing an opportunity to prevent future catastrophic declines associated with newly emergent disease via changes to our land management practices.

## Supporting Information

S1 Table ANOVA results examining treatment effects on average time to metamorphosis. **a.** Referent: Northern population, No shade, Acidified pH. b. Referent: Northern population, Shade, Acidified pH. c. Referent: Northern population, No Shade, Un-manipulated pH. d. Referent: Northern population, Shade, Un-manipulated pH. e. Referent: Southern population, No shade, Acidified pH. f. Referent: Southern population, Shade, Acidified pH. g. Referent: Southern population, No Shade, Un-manipulated pH. h. Referent: Southern population, Shade, Un-manipulated pH. Significant results in bold.
(DOCX)

S2 Table ANCOVA results examining treatment effects on Juvenile Mass. a. Referent: Northern population, No shade, Acidified pH. b. Referent: Northern population, Shade, Acidified pH. c. Referent: Northern population, No Shade, Un-manipulated pH. d. Referent: Northern population, Shade, Un-manipulated pH. e. Referent: Southern population, No shade, Acidified pH. f. Referent: Southern population, Shade, Acidified pH. g. Referent: Southern population, No Shade, Un-manipulated pH. h. Referent: Southern population, Shade, Un-manipulated pH. Significant results in bold.
(DOCX)

S3 Table ANCOVA results examining treatment effects on mean AMP production (standardized by gram body weight). a. Referent: Northern population, No shade, Acidified pH. b. Referent: Northern population, Shade, Acidified pH. c. Referent: Northern population, No Shade, Un-manipulated pH. d. Referent: Northern population, Shade, Un-manipulated pH. e. Referent: Southern population, No shade, Acidified pH. f. Referent: Southern population, Shade, Acidified pH. g. Referent: Southern population, No Shade, Un-manipulated pH. h. Referent: Southern population, Shade, Un-manipulated pH. Significant results in bold.
(DOCX)

S4 Table ANCOVA results examining treatment effects on AMP bioactivity (defined as the slope of the log-transformed growth curve). a. Referent: Northern population, No shade, Acidified pH. b. Referent: Northern population, Shade, Acidified pH. c. Referent: Northern population, No Shade, Un-manipulated pH. d. Referent: Northern population, Shade, Un-manipulated pH. e. Referent: Southern population, No shade, Acidified pH. f. Referent: Southern population, Shade, Acidified pH. g. Referent: Southern population, No Shade, Un-manipulated pH. h. Referent: Southern population, Shade, Un-manipulated pH. Significant results in bold.
(DOCX)

S5 Table ANCOVA results examining treatment effects on AMP bioactivity (defined as the Bd growth rate). a. Referent: Northern population, No shade, Acidified pH. b. Referent: Northern population, Shade, Acidified pH. c. Referent: Northern population, No Shade, Un-manipulated pH. d. Referent: Northern population, Shade, Un-manipulated pH. e. Referent: Southern population, No shade, Acidified pH. f. Referent: Southern population, Shade, Acidified pH. g. Referent: Southern population, No Shade, Un-manipulated pH. h. Referent: Southern population, Shade, Un-manipulated pH. Significant results in bold.
(DOCX)

S6 Table The sequence similarity of clones (out of 161 total) created from skin swabs of *R*.*catesbeiana* using primers 926r and 338f. Identification is based upon comparison to NCBI database entries using the FASTA program (National Center for Biotechnology Information). The percent identity (% ID) to best match is shown.
(DOCX)
