# Supplementary material for: Larval Environment Alters Amphibian Immune Defenses Differentially across Life Stages and Populations
Source: PLoS One. 2015 Jun 24;10(6):e0130383. doi: 10.1371/journal.pone.0130383 (PMC4479591; doi:10.1371/journal.pone.0130383)
Supplement: S1 Table — a. Referent: Northern population, No shade, Acidified pH. b. Referent: Northern population, Shade, Acidified pH. c. Referent: Northern population, No Shade, Un-manipulated pH. d. Referent: Northern population, Shade, Un-manipulated pH. e. Referent: Southern population, No shade, Acidified pH. f. Referent: Southern population, Shade, Acidified pH. g. Referent: Southern population, No Shade, Un-manipulated pH. h. Referent: Southern population, Shade, Un-manipulated pH. Significant results in bold. (DOCX) [file pone.0130383.s001.docx]

**S1 Table. ANOVA results examining treatment effects on average time to metamorphosis. a.** Referent: Northern population, No shade, Acidified pH. b. Referent: Northern population, Shade, Acidified pH. c. Referent: Northern population, No Shade, Un-manipulated pH. d. Referent: Northern population, Shade, Un-manipulated pH. e. Referent: Southern population, No shade, Acidified pH. f. Referent: Southern population, Shade, Acidified pH. g. Referent: Southern population, No Shade, Un-manipulated pH. h. Referent: Southern population, Shade, Un-manipulated pH. Significant results in bold.

**a. ANOVA results examining treatment effects on average time to metamorphosis.** Significant results in bold. Referent: Northern population, No shade, Acidified pH.

| **Response** | **Treatment** | **df** | **F** | **p** |
| --- | --- | --- | --- | --- |
| Larval Duration (days) | Acidification | 1,67 | 0.5121 | 0.4767 |
|  | **Shade** | **1,67** | **9.2956** | **0.0033** |
|  | **Population** | **1,67** | **11.6104** | **0.0011** |
|  | Block | 4,67 | 1.5853 | 0.1884 |

**b. ANOVA results examining treatment effects on average time to metamorphosis.** Significant results in bold. Referent: Northern population, Shade, Acidified pH.

| **Response** | **Treatment** | **df** | **F** | **p** |
| --- | --- | --- | --- | --- |
| Larval Duration (days) | Acidification | 1,67 | 0.5435 | 0.4636 |
|  | **Shade** | **1,67** | **9.2956** | **0.0033** |
|  | **Population** | **1,67** | **6.0467** | **0.0165** |
|  | Block | 4,67 | 1.5853 | 0.1884 |

**c. ANOVA results examining treatment effects on average time to metamorphosis.** Significant results in bold. Referent: Northern population, No Shade, Un-manipulated pH.

| **Response** | **Treatment** | **df** | **F** | **p** |
| --- | --- | --- | --- | --- |
| Larval Duration (days) | Acidification | 1,67 | 0.5121 | 0.4767 |
|  | **Shade** | **1,67** | **8.5333** | **0.0047** |
|  | **Population** | **1,67** | **17.2434** | **9.5x 10^-5^** |
|  | Block | 4,67 | 1.5853 | 0.1884 |

**d. ANOVA results examining treatment effects on average time to metamorphosis.** Significant results in bold. Referent: Northern population, Shade, Un-manipulated pH.

| **Response** | **Treatment** | **df** | **F** | **p** |
| --- | --- | --- | --- | --- |
| Larval Duration (days) | Acidification | 1,67 | 0.5435 | 0.4636 |
|  | **Shade** | **1,67** | **8.5333** | **0.0047** |
|  | **Population** | **1,67** | **13.8682** | **0.0004** |
|  | Block | 4,67 | 1.5853 | 0.1884 |

**e. ANOVA results examining treatment effects on average time to metamorphosis.** Significant results in bold. Referent: Southern population, No shade, Acidified pH.

| **Response** | **Treatment** | **df** | **F** | **p** |
| --- | --- | --- | --- | --- |
| Larval Duration (days) | Acidification | 1,67 | 0.0009 | 0.9766 |
|  | **Shade** | **1,67** | **4.4120** | **0.0395** |
|  | **Population** | **1,67** | **11.6104** | **0.0011** |
|  | Block | 4,67 | 1.5853 | 0.1884 |

**f. ANOVA results examining treatment effects on average time to metamorphosis.** Significant results in bold. Referent: Southern population, Shade, Acidified pH.

| **Response** | **Treatment** | **df** | **F** | **p** |
| --- | --- | --- | --- | --- |
| Larval Duration (days) | Acidification | 1,67 | 0.3774 | .54106 |
|  | **Shade** | **1,67** | **4.4120** | **0.0395** |
|  | **Population** | **1,67** | **6.0467** | **0.0165** |
|  | Block | 4,67 | 1.5853 | 0.1884 |

**g. ANOVA results examining treatment effects on average time to metamorphosis.** Significant results in bold. Referent: Southern population, No Shade, Un-manipulated pH.

| **Response** | **Treatment** | **df** | **F** | **p** |
| --- | --- | --- | --- | --- |
| Larval Duration (days) | Acidification | 1,67 | 0.0009 | 0.9766 |
|  | **Shade** | **1,67** | **7.2110** | **0.0091** |
|  | **Population** | **1,67** | **17.2434** | **9.5 x 10^-5^** |
|  | Block | 4,67 | 1.5853 | 0.1884 |

**h. ANOVA results examining treatment effects on average time to metamorphosis.** Significant results in bold. Referent: Southern population, Shade, Un-manipulated pH.

| **Response** | **Treatment** | **df** | **F** | **p** |
| --- | --- | --- | --- | --- |
| Larval Duration (days) | Acidification | 1,67 | 0.3774 | 0.5411 |
|  | **Shade** | **1,67** | **7.2110** | **0.0091** |
|  | **Population** | **1,67** | **13.8682** | **0.0004** |
|  | Block | 4,67 | 1.5853 | 0.1884 |
