# Supplementary material for: Larval Environment Alters Amphibian Immune Defenses Differentially across Life Stages and Populations
Source: PLoS One. 2015 Jun 24;10(6):e0130383. doi: 10.1371/journal.pone.0130383 (PMC4479591; doi:10.1371/journal.pone.0130383)
Supplement: S2 Table — a. Referent: Northern population, No shade, Acidified pH. b. Referent: Northern population, Shade, Acidified pH. c. Referent: Northern population, No Shade, Un-manipulated pH. d. Referent: Northern population, Shade, Un-manipulated pH. e. Referent: Southern population, No shade, Acidified pH. f. Referent: Southern population, Shade, Acidified pH. g. Referent: Southern population, No Shade, Un-manipulated pH. h. Referent: Southern population, Shade, Un-manipulated pH. Significant results in bold. (DOCX) [file pone.0130383.s002.docx]

**S2 Table. ANCOVA results examining treatment effects on Juvenile Mass.** a. Referent: Northern population, No shade, Acidified pH. b. Referent: Northern population, Shade, Acidified pH. c. Referent: Northern population, No Shade, Un-manipulated pH. d. Referent: Northern population, Shade, Un-manipulated pH. e. Referent: Southern population, No shade, Acidified pH. f. Referent: Southern population, Shade, Acidified pH. g. Referent: Southern population, No Shade, Un-manipulated pH. h. Referent: Southern population, Shade, Un-manipulated pH. Significant results in bold.

**a. ANCOVA results examining treatment effects on Juvenile Mass.** Significant results in bold. Referent: Northern population, No shade, Acidified pH.

| **Response** | **Treatment** | **df** | **F** | **p** |
| --- | --- | --- | --- | --- |
| Juvenile mass (g) | **Days in lab** | **1,65** | **8.7818** | **0.0042** |
|  | Acidification | 1,65 | 0.2705 | 0.6047 |
|  | Shade | 1,65 | .03182 | 0.5747 |
|  | **Population** | **1,65** | **7.6957** | **0.0072** |
|  | Block | 4,65 | 0.5596 | 0.6928 |

**b. ANCOVA results examining treatment effects on Juvenile Mass.** Significant results in bold. Referent: Northern population, Shade, Acidified pH.

| **Response** | **Treatment** | **df** | **F** | **p** |
| --- | --- | --- | --- | --- |
| Juvenile mass (g) | **Days in lab** | **1,65** | **8.7818** | **0.0042** |
|  | Acidification | 1,65 | 0.0174 | 0.8953 |
|  | Shade | 1,65 | 0.3182 | 0.5747 |
|  | Population | 1,65 | 3.0282 | 0.0866 |
|  | Block | 4,65 | 0.5596 | 0.6928 |

**c. ANCOVA results examining treatment effects on Juvenile Mass.** Significant results in bold. Referent: Northern population, No Shade, Un-manipulated pH.

| **Response** | **Treatment** | **df** | **F** | **p** |
| --- | --- | --- | --- | --- |
| Juvenile mass (g) | **Days in lab** | **1,65** | **8.7818** | **0.0042** |
|  | Acidification | 1,65 | 0.2705 | 0.6047 |
|  | Shade | 1,65 | 1.3172 | 0.2553 |
|  | **Population** | **1,65** | **5.6603** | **0.0203** |
|  | Block | 4,65 | 0.5596 | 0.6928 |

**d. ANCOVA results examining treatment effects on Juvenile Mass.** Significant results in bold. Referent: Northern population, Shade, Un-manipulated pH.

| **Response** | **Treatment** | **df** | **F** | **p** |
| --- | --- | --- | --- | --- |
| Juvenile mass (g) | **Days in lab** | **1,65** | **8.7818** | **0.0042** |
|  | Acidification | 1,65 | 0.0174 | 0.8953 |
|  | Shade | 1,65 | 1.3172 | 0.2553 |
|  | Population | 1,65 | 3.6277 | 0.0613 |
|  | Block | 4,65 | 0.5596 | 0.6928 |

**e. ANCOVA results examining treatment effects on Juvenile Mass.** Significant results in bold. Referent: Southern population, No shade, Acidified pH.

| **Response** | **Treatment** | **df** | **F** | **p** |
| --- | --- | --- | --- | --- |
| Juvenile mass (g) | **Days in lab** | **1,65** | **8.7818** | **0.0042** |
|  | Acidification | 1,65 | 0.6942 | 0.4078 |
|  | Shade | 1,65 | 0.1811 | 0.6718 |
|  | **Population** | **1,65** | **7.6957** | **0.0072** |
|  | Block | 4,65 | 0.5596 | 0.6928 |

**f. ANCOVA results examining treatment effects on Juvenile Mass.** Significant results in bold. Referent: Southern population, Shade, Acidified pH.

| **Response** | **Treatment** | **df** | **F** | **p** |
| --- | --- | --- | --- | --- |
| Juvenile mass (g) | **Days in lab** | **1,65** | **8.7818** | **0.0042** |
|  | Acidification | 1,65 | 0.1644 | 0.6865 |
|  | Shade | 1,65 | 0.1811 | 0.6718 |
|  | Population | 1,65 | 3.0282 | 0.0866 |
|  | Block | 4,65 | 0.5596 | 0.6928 |

**g. ANCOVA results examining treatment effects on Juvenile Mass.** Significant results in bold. Referent: Southern population, No Shade, Un-manipulated pH.

| **Response** | **Treatment** | **df** | **F** | **p** |
| --- | --- | --- | --- | --- |
| Juvenile mass (g) | **Days in lab** | **1,65** | **8.7818** | **0.0042** |
|  | Acidification | 1,65 | 0.6942 | 0.4078 |
|  | Shade | 1,65 | 0.4909 | 0.4860 |
|  | **Population** | **1,65** | **5.6603** | **0.0203** |
|  | Block | 4,65 | 0.5596 | 0.6928 |

**h. ANCOVA results examining treatment effects on Juvenile Mass.** Significant results in bold. Referent: Southern population, Shade, Un-manipulated pH.

| **Response** | **Treatment** | **df** | **F** | **p** |
| --- | --- | --- | --- | --- |
| Juvenile mass (g) | **Days in lab** | **1,65** | **8.7818** | **0.0042** |
|  | Acidification | 1,65 | 0.1644 | 0.6865 |
|  | Shade | 1,65 | 0.4909 | 0.4860 |
|  | Population | 1,65 | 3.6277 | 0.0613 |
|  | Block | 4,65 | 0.5596 | 0.6928 |
