# Supplementary material for: Larval Environment Alters Amphibian Immune Defenses Differentially across Life Stages and Populations
Source: PLoS One. 2015 Jun 24;10(6):e0130383. doi: 10.1371/journal.pone.0130383 (PMC4479591; doi:10.1371/journal.pone.0130383)
Supplement: S3 Table — a. Referent: Northern population, No shade, Acidified pH. b. Referent: Northern population, Shade, Acidified pH. c. Referent: Northern population, No Shade, Un-manipulated pH. d. Referent: Northern population, Shade, Un-manipulated pH. e. Referent: Southern population, No shade, Acidified pH. f. Referent: Southern population, Shade, Acidified pH. g. Referent: Southern population, No Shade, Un-manipulated pH. h. Referent: Southern population, Shade, Un-manipulated pH. Significant results in bold. (DOCX) [file pone.0130383.s003.docx]

**S3 Table. ANCOVA results examining treatment effects on mean AMP production (standardized by gram body weight).** a. Referent: Northern population, No shade, Acidified pH. b. Referent: Northern population, Shade, Acidified pH. c. Referent: Northern population, No Shade, Un-manipulated pH. d. Referent: Northern population, Shade, Un-manipulated pH.

e. Referent: Southern population, No shade, Acidified pH. f. Referent: Southern population, Shade, Acidified pH. g. Referent: Southern population, No Shade, Un-manipulated pH. h. Referent: Southern population, Shade, Un-manipulated pH. Significant results in bold.

**a. ANCOVA results examining treatment effects on mean AMP production (standardized by gram body weight).** Significant results in bold. Referent: Northern population, No shade, Acidified pH.

| **Response** | **Treatment** | **df** | **F** | **p** |
| --- | --- | --- | --- | --- |
| Mean AMP production (ug/ml) | Days in lab | 1,65 | 0.4087 | 0.5249 |
|  | **Acidification** | **1,65** | **3.9923** | **0.0499** |
|  | **Shade** | **1,65** | **4.1441** | **0.0459** |
|  | Population | 1,65 | 0.5392 | 0.4654 |
|  | Block | 4,65 | 0.4132 | 0.7985 |
|  | **Acid x Shade** | **1,65** | **5.1084** | **0.0272** |
|  | Acid x Population | 1,65 | 0.3452 | 0.5589 |
|  | **Shade x Population** | **1,65** | **3.9849** | **0.0501** |
|  | Acid x Shade x Population | 1,65 | 1.4104 | 0.2393 |
|  |  |  |  |  |

**b. ANCOVA results examining treatment effects on mean AMP production (standardized by gram body weight).** Significant results in bold. Referent: Northern population, Shade, Acidified pH.

| **Response** | **Treatment** | **df** | **F** | **p** |
| --- | --- | --- | --- | --- |
| Mean AMP production (ug/ml) | Days in lab | 1,65 | 0.4087 | 0.5249 |
|  | Acidification | 1,65 | 1.4242 | 0.2371 |
|  | **Shade** | **1,65** | **4.1441** | **0.0459** |
|  | **Population** | **1,65** | **4.3546** | **0.0408** |
|  | Block | 4,65 | 0.4132 | 0.7985 |
|  | **Acid x Shade** | **1,65** | **5.1084** | **0.0272** |
|  | Acid x Population | 1,65 | 1.2005 | 0.2773 |
|  | **Shade x Population** | **1,65** | **3.9849** | **0.0501** |
|  | Acid x Shade x Population | 1,65 | 1.4104 | 0.2393 |

**c. ANCOVA results examining treatment effects on mean AMP production (standardized by gram body weight).** Significant results in bold. Referent: Northern population, No Shade, Un-manipulated pH.

| **Response** | **Treatment** | **df** | **F** | **p** |
| --- | --- | --- | --- | --- |
| Mean AMP production (ug/ml) | Days in lab | 1,65 | 0.4087 | 0.5249 |
|  | **Acidification** | **1,65** | **3.9923** | **0.0499** |
|  | Shade | 1,65 | 0.8808 | 0.3515 |
|  | Population | 1,65 | 0.0100 | 0.9205 |
|  | Block | 4,65 | 0.4132 | 0.7985 |
|  | **Acid x Shade** | **1,65** | **5.1084** | **0.0272** |
|  | Acid x Population | 1,65 | 0.3452 | 0.5589 |
|  | Shade x Population | 1,65 | 0.0738 | 0.7868 |
|  | Acid x Shade x Population | 1,65 | 1.4104 | 0.2393 |

**d. ANCOVA results examining treatment effects on mean AMP production (standardized by gram body weight).** Significant results in bold. Referent: Northern population, Shade, Un-manipulated pH.

| **Response** | **Treatment** | **df** | **F** | **p** |
| --- | --- | --- | --- | --- |
| Mean AMP production (ug/ml) | Days in lab | 1,65 | 0.4087 | 0.5249 |
|  | Acidification | 1,65 | 1.4242 | 0.2371 |
|  | Shade | 1,65 | 0.8808 | 0.3515 |
|  | Population | 1,65 | 0.2252 | 0.6367 |
|  | Block | 4,65 | 0.4132 | 0.7985 |
|  | Acid x Shade | 1,65 | 5.1084 | 0.0272 |
|  | Acid x Population | 1,65 | 1.2005 | 0.27726 |
|  | Shade x Population | 1,65 | 0.0738 | 0.7868 |
|  | Acid x Shade x Population | 1,65 | 1.4104 | 0.2393 |

**e. ANCOVA results examining treatment effects on mean AMP production (standardized by gram body weight).** Significant results in bold. Referent: Southern population, No shade, Acidified pH.

| **Response** | **Treatment** | **df** | **F** | **p** |
| --- | --- | --- | --- | --- |
| Mean AMP production (ug/ml) | Days in lab | 1,65 | 0.4087 | 0.5249 |
|  | Acidification | 1,65 | 1.2432 | 0.2690 |
|  | Shade | 1,65 | 0.3543 | 0.5538 |
|  | Population | 1,65 | 0.5392 | 0.4654 |
|  | Block | 4,65 | 0.4132 | 0.7985 |
|  | Acid x Shade | 1,65 | 0.3094 | 0.5799 |
|  | Acid x Population | 1,65 | 0.3452 | 0.5589 |
|  | **Shade x Population** | **1,65** | **3.9849** | **0.0501** |
|  | Acid x Shade x Population | 1,65 | 1.4104 | 0.2393 |

**f. ANCOVA results examining treatment effects on mean AMP production (standardized by gram body weight).** Significant results in bold. Referent: Southern population, Shade, Acidified pH.

| **Response** | **Treatment** | **df** | **F** | **p** |
| --- | --- | --- | --- | --- |
| Mean AMP production (ug/ml) | Days in lab | 1,65 | 0.4087 | 0.5249 |
|  | Acidification | 1,65 | 0.1231 | 0.7268 |
|  | Shade | 1,65 | 0.3543 | 0.5538 |
|  | **Population** | **1,65** | **4.3546** | **0.0408** |
|  | Block | 4,65 | 0.4132 | 0.7985 |
|  | Acid x Shade | 1,65 | 0.3094 | 0.5710 |
|  | Acid x Population | 1,65 | 1.2005 | 0.2773 |
|  | **Shade x Population** | **1,65** | **3.9849** | **0.0501** |
|  | Acid x Shade x Population | 1,65 | 1.4104 | 0.2393 |

**g. ANCOVA results examining treatment effects on mean AMP production (standardized by gram body weight).** Significant results in bold. Referent: Southern population, No Shade, Un-manipulated pH.

| **Response** | **Treatment** | **df** | **F** | **p** |
| --- | --- | --- | --- | --- |
| Mean AMP production (ug/ml) | Days in lab | 1,65 | 0.4087 | 0.5249 |
|  | Acidification | 1,65 | 1.2432 | 0.2690 |
|  | Shade | 1,65 | 1.4712 | 0.2296 |
|  | Population | 1,65 | 0.0100 | 0.9205 |
|  | Block | 4,65 | 0.4132 | 0.7985 |
|  | Acid x Shade | 1,65 | 0.3094 | 0.5799 |
|  | Acid x Population | 1,65 | 0.3452 | 0.5589 |
|  | Shade x Population | 1,65 | 0.0738 | 0.7868 |
|  | Acid x Shade x Population | 1,65 | 1.4104 | 0.2393 |

**h. ANCOVA results examining treatment effects on mean AMP production (standardized by gram body weight).** Significant results in bold. Referent: Southern population, Shade, Un-manipulated pH.

| **Response** | **Treatment** | **df** | **F** | **p** |
| --- | --- | --- | --- | --- |
| Mean AMP production (ug/ml) | Days in lab | 1,65 | 0.4087 | 0.5249 |
|  | Acidification | 1,65 | 0.1231 | 0.7268 |
|  | Shade | 1,65 | 1.4712 | 0.2296 |
|  | Population | 1,65 | 0.2252 | 0.6367 |
|  | Block | 4,65 | 0.4132 | 0.7985 |
|  | Acid x Shade | 1,65 | 0.3094 | 0.5799 |
|  | Acid x Population | 1,65 | 1.2005 | 0.2773 |
|  | Shade x Population | 1,65 | 0.0738 | 0.7868 |
|  | Acid x Shade x Population | 1,65 | 1.4104 | 0.2393 |
