# Supplementary material for: Larval Environment Alters Amphibian Immune Defenses Differentially across Life Stages and Populations
Source: PLoS One. 2015 Jun 24;10(6):e0130383. doi: 10.1371/journal.pone.0130383 (PMC4479591; doi:10.1371/journal.pone.0130383)
Supplement: S4 Table — a. Referent: Northern population, No shade, Acidified pH. b. Referent: Northern population, Shade, Acidified pH. c. Referent: Northern population, No Shade, Un-manipulated pH. d. Referent: Northern population, Shade, Un-manipulated pH. e. Referent: Southern population, No shade, Acidified pH. f. Referent: Southern population, Shade, Acidified pH. g. Referent: Southern population, No Shade, Un-manipulated pH. h. Referent: Southern population, Shade, Un-manipulated pH. Significant results in bold. (DOCX) [file pone.0130383.s004.docx]

**S4 Table. ANCOVA results examining treatment effects on AMP bioactivity (defined as the slope of the log-transformed growth curve).** a. Referent: Northern population, No shade, Acidified pH. b. Referent: Northern population, Shade, Acidified pH. c. Referent: Northern population, No Shade, Un-manipulated pH. d. Referent: Northern population, Shade, Un-manipulated pH. e. Referent: Southern population, No shade, Acidified pH. f. Referent: Southern population, Shade, Acidified pH. g. Referent: Southern population, No Shade, Un-manipulated pH. h. Referent: Southern population, Shade, Un-manipulated pH. Significant results in bold.

**a. ANCOVA results examining treatment effects on AMP bioactivity (defined as the slope of the log-transformed growth curve).** Significant results in bold. Referent: Northern population, No shade, Acidified pH.

| **Response** | **Treatment** | **df** | **F** | **p** |
| --- | --- | --- | --- | --- |
| Bioactivity (slope) | Days in lab | 1,65 | 2.9846 | 0.0888 |
|  | Acidification | 1,65 | 1.1534 | 0.2868 |
|  | Shade | 1,65 | 0.9599 | 0.3309 |
|  | Population | 1,65 | 0.1112 | 0.7399 |
|  | Block | 4,65 | 0.8954 | 0.4719 |
|  | Acid x Shade | 1,65 | 1.3047 | 0.2575 |
|  | Acid x Population | 1,65 | 1.9464 | 0.1677 |
|  | Shade x Population | 1,65 | 0.2254 | 0.6365 |
|  | Acid x Shade x Population | 1,65 | 2.4998 | 0.1187 |
|  |  |  |  |  |

**b. ANCOVA results examining treatment effects on AMP bioactivity (defined as the slope of the log-transformed growth curve).** Significant results in bold. Referent: Northern population, Shade, Acidified pH.

| **Response** | **Treatment** | **df** | **F** | **p** |
| --- | --- | --- | --- | --- |
| Bioactivity (slope) | Days in lab | 1,65 | 2.9846 | 0.0888 |
|  | Acidification | 1,65 | 0.2925 | 0.5905 |
|  | Shade | 1,65 | 0.9599 | 0.3309 |
|  | Population | 1,65 | 0.1131 | 0.7378 |
|  | Block | 4,65 | 0.8954 | 0.4719 |
|  | Acid x Shade | 1,65 | 1.3047 | 0.2575 |
|  | Acid x Population | 1,65 | 0.7236 | 0.3981 |
|  | Shade x Population | 1,65 | 0.2254 | 0.6365 |
|  | Acid x Shade x Population | 1,65 | 2.4998 | 0.1187 |

**c. ANCOVA results examining treatment effects on AMP bioactivity (defined as the slope of the log-transformed growth curve).** Significant results in bold. Referent: Northern population, No Shade, Un-manipulated pH.

| **Response** | **Treatment** | **df** | **F** | **p** |
| --- | --- | --- | --- | --- |
| Bioactivity (slope) | Days in lab | 1,65 | 2.9846 | 0.0888 |
|  | Acidification | 1,65 | 1.1534 | 0.2868 |
|  | **Shade** | **1,65** | **5.9510** | **0.0175** |
|  | Population | 1,65 | 2.6192 | 0.1104 |
|  | Block | 4,65 | 0.8954 | 0.4719 |
|  | Acid x Shade | 1,65 | 1.3047 | 0.2575 |
|  | Acid x Population | 1,65 | 1.9464 | 0.1677 |
|  | Shade x Population | 1,65 | 3.0599 | 0.0850 |
|  | Acid x Shade x Population | 1,65 | 2.4998 | 0.1187 |

**d. ANCOVA results examining treatment effects on AMP bioactivity (defined as the slope of the log-transformed growth curve).** Significant results in bold. Referent: Northern population, Shade, Un-manipulated pH.

| **Response** | **Treatment** | **df** | **F** | **p** |
| --- | --- | --- | --- | --- |
| Bioactivity (slope) | Days in lab | 1,65 | 2.9846 | 0.0888 |
|  | Acidification | 1,65 | 0.2925 | 0.5905 |
|  | **Shade** | **1,65** | **5.9510** | **0.0175** |
|  | Population | 1,65 | 0.7188 | 0.3996 |
|  | Block | 4,65 | 0.8954 | 0.4719 |
|  | Acid x Shade | 1,65 | 1.3047 | 0.2575 |
|  | Acid x Population | 1,65 | 0.7236 | 0.3981 |
|  | Shade x Population | 1,65 | 3.0599 | 0.0850 |
|  | Acid x Shade x Population | 1,65 | 2.4998 | 0.1187 |

**e. ANCOVA results examining treatment effects on AMP bioactivity (defined as the slope of the log-transformed growth curve).** Significant results in bold. Referent: Southern population, No shade, Acidified pH.

| **Response** | **Treatment** | **df** | **F** | **p** |
| --- | --- | --- | --- | --- |
| Bioactivity (slope) | Days in lab | 1,65 | 2.9846 | 0.0888 |
|  | Acidification | 1,65 | 0.7853 | 0.3788 |
|  | Shade | 1,65 | 2.7261 | 0.1036 |
|  | Population | 1,65 | 0.1112 | 0.7399 |
|  | Block | 4,65 | 0.8954 | 0.4719 |
|  | Acid x Shade | 1,65 | 1.1987 | 0.2776 |
|  | Acid x Population | 1,65 | 1.9464 | 0.1677 |
|  | Shade x Population | 1,65 | 0.2254 | 0.6365 |
|  | Acid x Shade x Population | 1,65 | 2.4998 | 0.1187 |

**f. ANCOVA results examining treatment effects on AMP bioactivity (defined as the slope of the log-transformed growth curve).** Significant results in bold. Referent: Southern population, Shade, Acidified pH.

| **Response** | **Treatment** | **df** | **F** | **P** |
| --- | --- | --- | --- | --- |
| Bioactivity (slope) | Days in lab | 1,65 | 2.9846 | 0.0888 |
|  | Acidification | 1,65 | 0.4488 | 0.5053 |
|  | Shade | 1,65 | 2.7261 | 0.1036 |
|  | Population | 1,65 | 0.1131 | 0.7378 |
|  | Block | 4,65 | 0.8954 | 0.4719 |
|  | Acid x Shade | 1,65 | 1.1987 | 0.2776 |
|  | Acid x Population | 1,65 | 0.7236 | 0.3981 |
|  | Shade x Population | 1,65 | 0.2254 | 0.6351 |
|  | Acid x Shade x Population | 1,65 | 2.4998 | 0.1187 |

**g. ANCOVA results examining treatment effects on AMP bioactivity (defined as the slope of the log-transformed growth curve).** Significant results in bold. Referent: Southern population, No Shade, Un-manipulated pH.

| **Response** | **Treatment** | **df** | **F** | **p** |
| --- | --- | --- | --- | --- |
| Bioactivity (slope) | Days in lab | 1,65 | 2.9846 | 0.0888 |
|  | Acidification | 1,65 | 0.7853 | 0.3788 |
|  | Shade | 1,65 | 0.0132 | 0.9089 |
|  | Population | 1,65 | 2.6192 | 0.1104 |
|  | Block | 4,65 | 0.8954 | 0.4719 |
|  | Acid x Shade | 1,65 | 1.1987 | 0.2776 |
|  | Acid x Population | 1,65 | 1.9464 | 0.1677 |
|  | Shade x Population | 1,65 | 3.0599 | 0.0850 |
|  | Acid x Shade x Population | 1,65 | 2.4998 | 0.1187 |

**h. ANCOVA results examining treatment effects on AMP bioactivity (defined as the slope of the log-transformed growth curve).** Significant results in bold. Referent: Southern population, Shade, Un-manipulated pH.

| **Response** | **Treatment** | **df** | **F** | **P** |
| --- | --- | --- | --- | --- |
| Bioactivity (slope) | Days in lab | 1,65 | 2.9846 | 0.0888 |
|  | Acidification | 1,65 | 0.4488 | 0.5053 |
|  | Shade | 1,65 | 0.0132 | 0.9089 |
|  | Population | 1,65 | 0.7188 | 0.3996 |
|  | Block | 4,65 | 0.8954 | 0.4719 |
|  | Acid x Shade | 1,65 | 1.1987 | 0.2776 |
|  | Acid x Population | 1,65 | 0.7236 | 0.3981 |
|  | Shade x Population | 1,65 | 3.0599 | 0.0850 |
|  | Acid x Shade x Population | 1,65 | 2.4998 | 0.1187 |
