# Supplementary material for: Larval Environment Alters Amphibian Immune Defenses Differentially across Life Stages and Populations
Source: PLoS One. 2015 Jun 24;10(6):e0130383. doi: 10.1371/journal.pone.0130383 (PMC4479591; doi:10.1371/journal.pone.0130383)
Supplement: S5 Table — a. Referent: Northern population, No shade, Acidified pH. b. Referent: Northern population, Shade, Acidified pH. c. Referent: Northern population, No Shade, Un-manipulated pH. d. Referent: Northern population, Shade, Un-manipulated pH. e. Referent: Southern population, No shade, Acidified pH. f. Referent: Southern population, Shade, Acidified pH. g. Referent: Southern population, No Shade, Un-manipulated pH. h. Referent: Southern population, Shade, Un-manipulated pH. Significant results in bold. (DOCX) [file pone.0130383.s005.docx]

**S5 Table. ANCOVA results examining treatment effects on AMP bioactivity (defined as the Bd growth rate).** a. Referent: Northern population, No shade, Acidified pH. b. Referent: Northern population, Shade, Acidified pH. c. Referent: Northern population, No Shade, Un-manipulated pH. d. Referent: Northern population, Shade, Un-manipulated pH. e. Referent: Southern population, No shade, Acidified pH. f. Referent: Southern population, Shade, Acidified pH. g. Referent: Southern population, No Shade, Un-manipulated pH. h. Referent: Southern population, Shade, Un-manipulated pH. Significant results in bold.

**a. ANCOVA results examining treatment effects on AMP bioactivity (defined as the Bd growth rate).** Significant results in bold. Referent: Northern population, No shade, Acidified pH.

| **Response** | **Treatment** | **df** | **F** | **p** |
| --- | --- | --- | --- | --- |
| Bioactivity (Bd growth rate) | Days in lab | 1,65 | 2.6786 | 0.1065 |
|  | **Acidification** | **1,65** | **4.3112** | **0.0418** |
|  | Shade | 1,65 | 0.3013 | 0.5850 |
|  | Population | 1,65 | 1.4540 | 0.2323 |
|  | Block | 4,65 | 0.2306 | 0.9202 |
|  | Acid x Shade | 1,65 | 0.3860 | 0.5366 |
|  | **Acid x Population** | **1,65** | **4.7649** | **0.0327** |
|  | Shade x Population | 1,65 | 0.0112 | 0.9159 |
|  | Acid x Shade x Population | 1,65 | 0.0840 | 0.7729 |
|  |  |  |  |  |

**b. ANCOVA results examining treatment effects on AMP bioactivity (defined as the Bd growth rate).** Significant results in bold. Referent: Northern population, Shade, Acidified pH.

| **Response** | **Treatment** | **df** | **F** | **p** |
| --- | --- | --- | --- | --- |
| Bioactivity (Bd growth rate) | Days in lab | 1,65 | 2.6786 | 0.1065 |
|  | Acidification | 1,65 | 1.3347 | 0.2522 |
|  | Shade | 1,65 | 0.3013 | 0.5850 |
|  | Population | 1,65 | 1.8659 | 0.1767 |
|  | Block | 4,65 | 0.2306 | 0.9202 |
|  | Acid x Shade | 1,65 | 0.3860 | 0.5366 |
|  | Acid x Population | 1,65 | 3.0824 | 0.0839 |
|  | Shade x Population | 1,65 | 0.0112 | 0.9159 |
|  | Acid x Shade x Population | 1,65 | 0.0840 | 0.7729 |

**c. ANCOVA results examining treatment effects on AMP bioactivity (defined as the Bd growth rate).** Significant results in bold. Referent: Northern population, No Shade, Un-manipulated pH.

| **Response** | **Treatment** | **df** | **F** | **p** |
| --- | --- | --- | --- | --- |
| Bioactivity (Bd growth rate) | Days in lab | 1,65 | 2.6786 | 0.1065 |
|  | **Acidification** | **1,65** | **4.3112** | **0.0418** |
|  | Shade | 1,65 | 1.8023 | 0.1841 |
|  | Population | 1,65 | 3.4687 | 0.0671 |
|  | Block | 4,65 | 0.2306 | 0.9202 |
|  | Acid x Shade | 1,65 | 0.3860 | 0.5366 |
|  | **Acid x Population** | **1,65** | **4.7649** | **0.0327** |
|  | Shade x Population | 1,65 | 0.2574 | 0.6136 |
|  | Acid x Shade x Population | 1,65 | 0.0840 | 0.7729 |

**d. ANCOVA results examining treatment effects on AMP bioactivity (defined as the Bd growth rate).** Significant results in bold. Referent: Northern population, Shade, Un-manipulated pH.

| **Response** | **Treatment** | **df** | **F** | **p** |
| --- | --- | --- | --- | --- |
| Bioactivity (Bd growth rate) | Days in lab | 1,65 | 2.6786 | 0.1065 |
|  | Acidification | 1,65 | 1.3347 | 0.2522 |
|  | Shade | 1,65 | 1.8023 | 0.1841 |
|  | Population | 1,65 | 1.2395 | 0.2697 |
|  | Block | 4,65 | 0.2306 | 0.9202 |
|  | Acid x Shade | 1,65 | 0.3860 | 0.5366 |
|  | Acid x Population | 1,65 | 3.0824 | 0.0839 |
|  | Shade x Population | 1,65 | 0.2574 | 0.6136 |
|  | Acid x Shade x Population | 1,65 | 0.0840 | 0.7729 |

**e. ANCOVA results examining treatment effects on AMP bioactivity (defined as the Bd growth rate).** Significant results in bold. Referent: Southern population, No shade, Acidified pH.

| **Response** | **Treatment** | **df** | **F** | **p** |
| --- | --- | --- | --- | --- |
| Bioactivity (Bd growth rate) | Days in lab | 1,65 | 2.6786 | 0.1065 |
|  | Acidification | 1,65 | 1.0106 | 0.3185 |
|  | Shade | 1,65 | 0.1788 | 0.6738 |
|  | Population | 1,65 | 1.4540 | 0.2323 |
|  | Block | 4,65 | 0.2306 | 0.9202 |
|  | Acid x Shade | 1,65 | 0.0417 | 0.8388 |
|  | **Acid x Population** | **1,65** | **4.7649** | **0.0327** |
|  | Shade x Population | 1,65 | 0.0112 | 0.9159 |
|  | Acid x Shade x Population | 1,65 | 0.0840 | 0.7729 |

**f. ANCOVA results examining treatment effects on AMP bioactivity (defined as the Bd growth rate).** Significant results in bold. Referent: Southern population, Shade, Acidified pH.

| **Response** | **Treatment** | **df** | **F** | **P** |
| --- | --- | --- | --- | --- |
| Bioactivity (Bd growth rate) | Days in lab | 1,65 | 2.6786 | 0.1065 |
|  | Acidification | 1,65 | 1.8018 | 0.1842 |
|  | Shade | 1,65 | 0.1788 | 0.6738 |
|  | Population | 1,65 | 1.8659 | 0.1767 |
|  | Block | 4,65 | 0.2306 | 0.9202 |
|  | Acid x Shade | 1,65 | 0.0417 | 0.8388 |
|  | Acid x Population | 1,65 | 3.0824 | 0.0839 |
|  | Shade x Population | 1,65 | 0.0112 | 0.9159 |
|  | Acid x Shade x Population | 1,65 | 0.0840 | 0.7729 |

**g. ANCOVA results examining treatment effects on AMP bioactivity (defined as the Bd growth rate).** Significant results in bold. Referent: Southern population, No Shade, Un-manipulated pH.

| **Response** | **Treatment** | **df** | **F** | **p** |
| --- | --- | --- | --- | --- |
| Bioactivity (Bd growth rate) | Days in lab | 1,65 | 2.6786 | 0.1065 |
|  | Acidification | 1,65 | 1.0106 | 0.3185 |
|  | Shade | 1,65 | 0.36926 | 0.5331 |
|  | Population | 1,65 | 3.4687 | 0.0671 |
|  | Block | 4,65 | 0.2306 | 0.9202 |
|  | Acid x Shade | 1,65 | 0.0417 | 0.8388 |
|  | **Acid x Population** | **1,65** | **4.7649** | **0.0327** |
|  | Shade x Population | 1,65 | 0.2574 | 0.6136 |
|  | Acid x Shade x Population | 1,65 | 0.0840 | 0.7729 |

**h. ANCOVA results examining treatment effects on AMP bioactivity (defined as the Bd growth rate).** Significant results in bold. Referent: Southern population, Shade, Un-manipulated pH.

| **Response** | **Treatment** | **df** | **F** | **P** |
| --- | --- | --- | --- | --- |
| Bioactivity (Bd growth rate) | Days in lab | 1,65 | 2.6786 | 0.1065 |
|  | Acidification | 1,65 | 1.8018 | 0.1842 |
|  | Shade | 1,65 | 0.3926 | 0.5331 |
|  | Population | 1,65 | 1.2395 | 0.2697 |
|  | Block | 4,65 | 0.2306 | 0.9202 |
|  | Acid x Shade | 1,65 | 0.0417 | 0.8388 |
|  | Acid x Population | 1,65 | 3.0824 | 0.0839 |
|  | Shade x Population | 1,65 | 0.2574 | 0.6136 |
|  | Acid x Shade x Population | 1,65 | 0.0840 | 0.7729 |
