# Supplementary material for: Larval Environment Alters Amphibian Immune Defenses Differentially across Life Stages and Populations
Source: PLoS One. 2015 Jun 24;10(6):e0130383. doi: 10.1371/journal.pone.0130383 (PMC4479591; doi:10.1371/journal.pone.0130383)
Supplement: S6 Table — Identification is based upon comparison to NCBI database entries using the FASTA program (National Center for Biotechnology Information). The percent identity (% ID) to best match is shown. (DOCX) [file pone.0130383.s006.docx]

**S6 Table. The sequence similarity of clones (out of 161 total) created from skin swabs of *R.catesbeiana* using primers 926r and 338f.** Identification is based upon comparison to NCBI database entries using the FASTA program (National Center for Biotechnology Information). The percent identity (% ID) to best match is shown.

| **Clone ID** | **Clone Accession ID** | **Best Match**  **Accession ID** | **Best Match** | **%ID** | | **Division** |
| --- | --- | --- | --- | --- | --- | --- |
| BF_M01 | HF947349 | HE993549.1 | *Ideonella sp.* | 99 | | Betaproteobacteria |
| BF_M03 | HF947350 | [HE993549.2](http://www.ncbi.nlm.nih.gov/nucleotide/406356658?report=genbank&log$=nucltop&blast_rank=1&RID=J5KFRZFR013) | *Ideonella sp.* | 100 | | Betaproteobacteria |
| BF_M04 | HF947351 | [HE993549.2](http://www.ncbi.nlm.nih.gov/nucleotide/406356658?report=genbank&log$=nucltop&blast_rank=1&RID=J5KFRZFR013) | *Ideonella sp.* | 99 | | Betaproteobacteria |
| BF_M05 | HF947352 | [JX177698.1](http://www.ncbi.nlm.nih.gov/nucleotide/406822008?report=genbank&log$=nucltop&blast_rank=1&RID=J5KUCMCT015) | *Limnobacter sp.* | 99 | | Betaproteobacteria |
| BF_M06 | HF947353 | [HE614874.1](http://www.ncbi.nlm.nih.gov/nucleotide/358633468?report=genbank&log$=nucltop&blast_rank=1&RID=J5M3HGWJ015) | *Vogesella perlucida* | 99 | | Betaproteobacteria |
| BF_M07 | HF947354 | [HE653237.1](http://www.ncbi.nlm.nih.gov/nucleotide/373937977?report=genbank&log$=nucltop&blast_rank=1&RID=J5MDYUNJ01R) | *Flavobacterium sp.* | 99 | | Bacteriodetes |
| BF_M08 | HF947355 | [KC294078.1](http://www.ncbi.nlm.nih.gov/nucleotide/429844751?report=genbank&log$=nucltop&blast_rank=1&RID=J5MMP1T101R) | *Comamonas sp.* | 98 | | Betaproteobacteria |
| BF_M09 | HF947356 | [HE993549.2](http://www.ncbi.nlm.nih.gov/nucleotide/406356658?report=genbank&log$=nucltop&blast_rank=1&RID=J5KFRZFR013) | *Ideonella sp.* | 100 | | Betaproteobacteria |
| BF_M10 | HF947357 | [HE614874.1](http://www.ncbi.nlm.nih.gov/nucleotide/358633468?report=genbank&log$=nucltop&blast_rank=1&RID=J5M3HGWJ015) | *Vogesella perlucida* | 98 | | Betaproteobacteria |
| BF_M11 | HF947358 | [HE993549.2](http://www.ncbi.nlm.nih.gov/nucleotide/406356658?report=genbank&log$=nucltop&blast_rank=1&RID=J5KFRZFR013) | *Ideonella sp.* | 99 | | Betaproteobacteria |
| BF_M12 | HF947359 | [GQ284439.1](http://www.ncbi.nlm.nih.gov/nucleotide/254682091?report=genbank&log$=nucltop&blast_rank=3&RID=J78DC5MU013) | *Limnobacter thioxidans* | 96 | | Betaproteobacteria |
| BF_M13 | HF947360 | [HE993549.2](http://www.ncbi.nlm.nih.gov/nucleotide/406356658?report=genbank&log$=nucltop&blast_rank=1&RID=J5KFRZFR013) | *Ideonella sp.* | 100 | | Betaproteobacteria |
| BF_M14 | HF947361 | [HE993549.2](http://www.ncbi.nlm.nih.gov/nucleotide/406356658?report=genbank&log$=nucltop&blast_rank=1&RID=J5KFRZFR013) | *Ideonella sp.* | 100 | | Betaproteobacteria |
| BF_M15 | HF947362 | [HQ396921.1](http://www.ncbi.nlm.nih.gov/nucleotide/317159880?report=genbank&log$=nucltop&blast_rank=2&RID=J79K5WF1013) | *Acineto bacterjunii* | 100 | Gammaproteobacteria | |
| BF_M16 | HF947363 | [HE993549.2](http://www.ncbi.nlm.nih.gov/nucleotide/406356658?report=genbank&log$=nucltop&blast_rank=1&RID=J5KFRZFR013) | *Ideonella sp.* | 99 | | Betaproteobacteria |
| BF_M17 | HF947364 | [HE993549.2](http://www.ncbi.nlm.nih.gov/nucleotide/406356658?report=genbank&log$=nucltop&blast_rank=1&RID=J5KFRZFR013) | *Ideonella sp.* | 100 | | Betaproteobacteria |
| BF_M18 | HF947365 | [HE993549.2](http://www.ncbi.nlm.nih.gov/nucleotide/406356658?report=genbank&log$=nucltop&blast_rank=1&RID=J5KFRZFR013) | *Ideonella sp.* | 100 | | Betaproteobacteria |
| BF_M19 | HF947366 | [FM886888.1](http://www.ncbi.nlm.nih.gov/nucleotide/304939541?report=genbank&log$=nucltop&blast_rank=2&RID=J7CDUBVH01R) | *Pelomonas saccharophila* | 100 | | Betaproteobacteria |
| BF_M20 | HF947367 | [AB627080.1](http://www.ncbi.nlm.nih.gov/nucleotide/332144242?report=genbank&log$=nucltop&blast_rank=2&RID=J7CN6HEH01R) | *Clostrdium sensustricto* | 100 | | Firmicutes |
| BF_M22 | HF947368 | [GQ284439.1](http://www.ncbi.nlm.nih.gov/nucleotide/254682091?report=genbank&log$=nucltop&blast_rank=3&RID=JA2216F001R) | *Limnobacter thioxidans* | 98 | | Betaproteobacteria |
| BF_M23 | HF947369 | [KC294078.1](http://www.ncbi.nlm.nih.gov/nucleotide/429844751?report=genbank&log$=nucltop&blast_rank=1&RID=JA28FTYP013) | *Comamonas sp.* | 100 | | Betaproteobacteria |
| BF_M24 | HF947370 | [NR_043315.1](http://www.ncbi.nlm.nih.gov/nucleotide/343202830?report=genbank&log$=nucltop&blast_rank=2&RID=JA2J20CE01R) | *Brevundimonas kwangchunensis* | 100 | | Alphaproteobacteria |
| BF_M25 | HF947371 | [NR_044326.1](http://www.ncbi.nlm.nih.gov/nucleotide/343199019?report=genbank&log$=nucltop&blast_rank=2&RID=JA3C753W01R) | *Vogesella sp.* | 100 | | Betaproteobacteria |
| BF_M26 | HF947372 | [FM886864.1](http://www.ncbi.nlm.nih.gov/nucleotide/304854960?report=genbank&log$=nucltop&blast_rank=1&RID=JA3TTER3013) | *Comamonadaceae bacterium* | 99 | | Betaproteobacteria |
| BF_M28 | HF947373 | [HE993549.1](http://www.ncbi.nlm.nih.gov/nucleotide/406356658?report=genbank&log$=nucltop&blast_rank=1&RID=JA438TA4013) | *Ideonella sp.* | 99 | | Betaproteobacteria |
| BF_M29 | HF947374 | [GQ284439.1](http://www.ncbi.nlm.nih.gov/nucleotide/254682091?report=genbank&log$=nucltop&blast_rank=3&RID=JA4875Y801R) | *Limnobacter sp.* | 100 | | Betaproteobacteria |
| BF_M30 | HF947375 | [HE993549.1](http://www.ncbi.nlm.nih.gov/nucleotide/406356658?report=genbank&log$=nucltop&blast_rank=1&RID=JA438TA4013) | *Ideonella sp.* | 99 | | Betaproteobacteria |
| BF_M31 | HF947376 | [AY308840.1](http://www.ncbi.nlm.nih.gov/nucleotide/32251010?report=genbank&log$=nucltop&blast_rank=1&RID=KESK6FFC014) | *Flectobacillus sp.* | 99 | | Bacteriodetes |
| BF_M32 | HF947377 | [HE993549.1](http://www.ncbi.nlm.nih.gov/nucleotide/406356658?report=genbank&log$=nucltop&blast_rank=1&RID=JA438TA4013) | *Ideonella sp.* | 97 | | Betaproteobacteria |
| BF_M33 | HF947378 | HE614874.1 | *Vogesella perlucida* | 100 | | Betaproteobacteria |
| BF_M34 | HF947379 | [HE993549.1](http://www.ncbi.nlm.nih.gov/nucleotide/406356658?report=genbank&log$=nucltop&blast_rank=1&RID=JA438TA4013) | *Ideonella sp.* | 99 | | Betaproteobacteria |
| BF_M35 | HF947380 | [AJ556799.1](http://www.ncbi.nlm.nih.gov/nucleotide/30024214?report=genbank&log$=nucltop&blast_rank=1&RID=KEV5FEPC016) | *Comamonadaceae* | 98 | | Betaproteobacteria |
| BF_M36 | HF947381 | HE614874.1 | *Vogesella perlucida* | 99 | | Betaproteobacteria |
| BF_M37 | HF947382 | [JQ317253.1](http://www.ncbi.nlm.nih.gov/nucleotide/375073539?report=genbank&log$=nucltop&blast_rank=1&RID=KEVNMKDV014) | *Bacteriodes sp.* | 99 | | Bacteriodetes |
| BF_M38 | HF947383 | [GQ284439.1](http://www.ncbi.nlm.nih.gov/nucleotide/254682091?report=genbank&log$=nucltop&blast_rank=3&RID=KEW3H82801R) | *Limnobacter thioxidans* | 99 | | Betaproteobacteria |
| BF_M39 | HF947384 | [DQ854973.1](http://www.ncbi.nlm.nih.gov/nucleotide/112361421?report=genbank&log$=nucltop&blast_rank=1&RID=KEWA8WCE014) | *Ideonella sp.* | 93 | | Betaproteobacteria |
| BF_M40 | HF947385 | [GQ284439.1](http://www.ncbi.nlm.nih.gov/nucleotide/254682091?report=genbank&log$=nucltop&blast_rank=3&RID=KEWJ2YAX014) | *Limnobacter thioxidans* | 96 | | Betaproteobacteria |
| BF_M41 | HF947386 | [HE993549.1](http://www.ncbi.nlm.nih.gov/nucleotide/406356658?report=genbank&log$=nucltop&blast_rank=1&RID=JA438TA4013) | *Ideonella sp.* | 99 | | Betaproteobacteria |
| BF_M43 | HF947387 | [HE993549.1](http://www.ncbi.nlm.nih.gov/nucleotide/406356658?report=genbank&log$=nucltop&blast_rank=1&RID=JA438TA4013) | *Ideonella sp.* | 100 | | Betaproteobacteria |
| BF_M44 | HF947388 | [HE648174.1](http://www.ncbi.nlm.nih.gov/nucleotide/371501027?report=genbank&log$=nucltop&blast_rank=1&RID=KF9TP711016) | *Undibacterium sp.* | 99 | | Betaproteobacteria |
| BF_M45 | HF947389 | [M99574.1](http://www.ncbi.nlm.nih.gov/nucleotide/174506?report=genbank&log$=nucltop&blast_rank=1&RID=KFA1JD2K016) | *Epulopiscium sp.* | 99 | | Firmicutes |
| BF_M46 | HF947390 | [AB698738.1](http://www.ncbi.nlm.nih.gov/nucleotide/397787509?report=genbank&log$=nucltop&blast_rank=1&RID=KFAH1XGA01R) | *Methylotenera mobilis* | 99 | | Betaproteobacteria |
| BF_M47 | HF947391 | [HE993549.1](http://www.ncbi.nlm.nih.gov/nucleotide/406356658?report=genbank&log$=nucltop&blast_rank=1&RID=KFH0UJ55016) | *Ideonella sp.* | 99 | | Betaproteobacteria |
| BF_M48 | HF947392 | [JX177698.1](http://www.ncbi.nlm.nih.gov/nucleotide/406822008?report=genbank&log$=nucltop&blast_rank=2&RID=KFGDXYMJ014) | *Limnobacter sp.* | 88 | | Betaproteobacteria |
| BF_M49 | HF947393 | [GQ284439.1](http://www.ncbi.nlm.nih.gov/nucleotide/254682091?report=genbank&log$=nucltop&blast_rank=3&RID=KFGNKV1Y01R) | *Limnobacter thioxidans* | 97 | | Betaproteobacteria |
| BF_M50 | HF947394 | [HE993549.1](http://www.ncbi.nlm.nih.gov/nucleotide/406356658?report=genbank&log$=nucltop&blast_rank=1&RID=KFHDDP8J014) | *Ideonella sp.* | 99 | | Betaproteobacteria |
| BF_M51 | HF947395 | [HE614874.1](http://www.ncbi.nlm.nih.gov/nucleotide/358633468?report=genbank&log$=nucltop&blast_rank=1&RID=KM6A3X7U01R) | *Vogesella perlucida* | 99 | | Betaproteobacteria |
| BF_M52 | HF947396 | [HE993549.1](http://www.ncbi.nlm.nih.gov/nucleotide/406356658?report=genbank&log$=nucltop&blast_rank=1&RID=KM6JKRM6014) | *Ideonella sp.* | 99 | | Betaproteobacteria |
| BF_M53 | HF947397 | [HE993549.1](http://www.ncbi.nlm.nih.gov/nucleotide/406356658?report=genbank&log$=nucltop&blast_rank=1&RID=KM6U6CWB014) | *Ideonella sp.* | 97 | | Betaproteobacteria |
| BF_M55 | HF947398 | [HE614874.1](http://www.ncbi.nlm.nih.gov/nucleotide/358633468?report=genbank&log$=nucltop&blast_rank=1&RID=KM9WHCW8016) | *Vogesella perlucida* | 99 | | Betaproteobacteria |
| BF_M56 | HF947399 | [HE614874.1](http://www.ncbi.nlm.nih.gov/nucleotide/358633468?report=genbank&log$=nucltop&blast_rank=1&RID=KMA6UHJW01R) | *Vogesella perlucida* | 99 | | Betaproteobacteria |
| BF_M57 | HF947400 | [HE993549.1](http://www.ncbi.nlm.nih.gov/nucleotide/406356658?report=genbank&log$=nucltop&blast_rank=1&RID=KMAJRZZ001R) | *Ideonella sp.* | 99 | | Betaproteobacteria |
| BF_M58 | HF947401 | [HE614874.1](http://www.ncbi.nlm.nih.gov/nucleotide/358633468?report=genbank&log$=nucltop&blast_rank=1&RID=KMAV3PH1016) | *Vogesella perlucida* | 99 | | Betaproteobacteria |
| BF_M60 | HF947402 | [HE993549.1](http://www.ncbi.nlm.nih.gov/nucleotide/406356658?report=genbank&log$=nucltop&blast_rank=1&RID=KMB6PUXP01R) | *Ideonella sp.* | 99 | | Betaproteobacteria |
| BF_M61 | HF947403 | [HE993549.1](http://www.ncbi.nlm.nih.gov/nucleotide/406356658?report=genbank&log$=nucltop&blast_rank=1&RID=KMB6PUXP01R) | *Ideonella sp.* | 99 | | Betaproteobacteria |
| BF_M62 | HF947404 | [HE993549.1](http://www.ncbi.nlm.nih.gov/nucleotide/406356658?report=genbank&log$=nucltop&blast_rank=1&RID=KMB6PUXP01R) | *Ideonella sp.* | 99 | | Betaproteobacteria |
| BF_M63 | HF947405 | HE614874.1 | *Vogesella perlucida* | 96 | | Betaproteobacteria |
| BF_M65 | HF947406 | HE993549.1 | *Ideonella sp.* | 99 | | Betaproteobacteria |
| BF_M66 | HF947407 | HE614874.1 | *Vogesella perlucida* | 99 | | Betaproteobacteria |
| BF_M68 | HF947408 | AB076853.1 | *Comamonas sp.* | 99 | | Betaproteobacteria |
| BF_M70 | HF947409 | JX177698.1 | *Limnobacter sp.* | 97 | | Betaproteobacteria |
| BF_M71 | HF947410 | HE993549.1 | *Ideonella sp.* | 99 | | Betaproteobacteria |
| BF_M72 | HF947411 | HQ288929.1 | *Azospirillum lipoferum* | 99 | | Alphaproteobacteria |
| BF_M73 | HF947412 | FJ906694.2 | *Rhodobacter sp.* | 99 | | Alphaproteobacteria |
| BF_M74 | HF947413 | HE614874.1 | *Vogesella perlucida* | 99 | | Betaproteobacteria |
| BF_M75 | HF947414 | HQ538615.1 | *Herbaspirillum sp.* | 95 | | Betaproteobacteria |
| BF_M77 | HF947415 | HE614874.1 | *Vogesella perlucida* | 96 | | Betaproteobacteria |
| BF_M78 | HF947416 | HE993549.1 | *Ideonella sp.* | 99 | | Betaproteobacteria |
| BF_M79 | HF947417 | HE993549.1 | *Ideonella sp.* | 99 | | Betaproteobacteria |
| BF_M80 | HF947418 | HE993549.1 | *Ideonella sp.* | 99 | | Betaproteobacteria |
| BF_M82 | HF947419 | JX177698.1 | *Limnobacter sp.* | 99 | | Betaproteobacteria |
| BF_M83 | HF947420 | HE993549.1 | *Ideonella sp.* | 99 | | Betaproteobacteria |
| BF_M84 | HF947421 | HE993549.1 | *Ideonella sp.* | 99 | | Betaproteobacteria |
| BF_M85 | HF947422 | HE993549.1 | *Ideonella sp.* | 99 | | Betaproteobacteria |
| BF_M86 | HF947423 | JQ995475.1 | *Zoogloea resiniphila* | 96 | | Betaproteobacteria |
| BF_M87 | HF947424 | AB696863.1 | *Ideonella sp.* | 99 | | Betaproteobacteria |
| BF_M88 | HF947425 | HE614874.1 | *Vogesella perlucida* | 99 | | Betaproteobacteria |
| BF_M89 | HF947426 | HE993549.1 | *Ideonella sp.* | 99 | | Betaproteobacteria |
| BF_M90 | HF947427 | HE993549.1 | *Ideonella sp.* | 99 | | Betaproteobacteria |
| BF_M91 | HF947428 | HE616175.1 | *Rhizobacter sp.* | 97 | Gammaproteobacteria | |
| BF_M92 | HF947429 | HE614874.1 | *Vogesella perlucida* | 94 | | Betaproteobacteria |
| BF_M93 | HF947430 | HE993549.1 | *Ideonella sp.* | 99 | | Betaproteobacteria |
| BF_M94 | HF947431 | HE614874.1 | *Vogesella perlucida* | 99 | | Betaproteobacteria |
| BF_T01 | HF947432 | JF102672.1 | *Chitinophaga ginsengisegetis* | 91 | | Bacteriodetes |
| BF_T02 | HF947433 | HE600686.1 | *Limnohabitans sp.* | 99 | | Betaproteobacteria |
| BF_T03 | HF947434 | JF710262.1 | *Chitinophaga sp.* | 91 | | Bacteriodetes |
| BF_T04 | HF947435 | JF710262.1 | *Chitinophaga sp.* | 91 | | Bacteriodetes |
| BF_T05 | HF947436 | JF710262.1 | *Chitinophaga sp.* | 91 | | Bacteriodetes |
| BF_T08 | HF947437 | JF710262.1 | *Chitinophaga sp.* | 91 | | Bacteriodetes |
| BF_T09 | HF947438 | JF710262.1 | *Chitinophaga sp.* | 91 | | Bacteriodetes |
| BF_T11 | HF947439 | HE600686.1 | *Limnohabitans sp.* | 99 | | Betaproteobacteria |
| BF_T12 | HF947440 | HE600686.1 | *Limnohabitans sp.* | 99 | | Betaproteobacteria |
| BF_T13 | HF947441 | AB353123.1 | *Cetobacterium somerae* | 97 | | Fusobacteriales |
| BF_T14 | HF947442 | JF710262.1 | *Chitinophaga sp.* | 91 | | Bacteriodetes |
| BF_T16 | HF947443 | JF824804.1 | *Alistipes sp.* | 93 | | Bacteriodetes |
| BF_T17 | HF947444 | HE600686.1 | *Limnohabitans sp.* | 99 | | Betaproteobacteria |
| BF_T18 | HF947445 | HE600686.1 | *Limnohabitans sp.* | 98 | | Betaproteobacteria |
| BF_T19 | HF947446 | JF710262.1 | *Chitinophaga sp.* | 91 | | Bacteriodetes |
| BF_T20 | HF947447 | JF710262.1 | *Chitinophaga sp.* | 91 | | Bacteriodetes |
| BF_T22 | HF947448 | JF710262.1 | *Chitinophaga sp.* | 91 | | Bacteriodetes |
| BF_T23 | HF947449 | AB793710.1 | *Clostridium sp.* | 90 | | Firmicutes |
| BF_T24 | HF947450 | JF710262.1 | *Chitinophaga sp.* | 91 | | Bacteriodetes |
| BF_T25 | HF947451 | JF710262.1 | *Chitinophaga sp.* | 91 | | Bacteriodetes |
| BF_T27 | HF947452 | JF710262.1 | *Chitinophaga sp.* | 91 | | Bacteriodetes |
| BF_T28 | HF947453 | JF710262.1 | *Chitinophaga sp.* | 91 | | Bacteriodetes |
| BF_T29 | HF947454 | JF710262.1 | *Chitinophaga sp.* | 91 | | Bacteriodetes |
| BF_T30 | HF947455 | HE600686.1 | *Limnohabitans sp.* | 99 | | Betaproteobacteria |
| BF_T31 | HF947456 | JF710262.1 | *Chitinophaga sp.* | 91 | | Bacteriodetes |
| BF_T32 | HF947457 | JF710262.1 | *Chitinophaga sp.* | 91 | | Bacteriodetes |
| BF_T34 | HF947458 | JF824804.1 | *Alistipes sp.* | 92 | | Bacteriodetes |
| BF_T35 | HF947459 | JF824804.1 | *Alistipes sp.* | 92 | | Bacteriodetes |
| BF_T36 | HF947460 | JF710262.1 | *Chitinophaga sp.* | 91 | | Bacteriodetes |
| BF_T37 | HF947461 | JF710262.1 | *Chitinophaga sp.* | 91 | | Bacteriodetes |
| BF_T39 | HF947462 | NR 025421.1 | *Limnobacter thiooxidans* | 91 | | Betaproteobacteria |
| BF_T40 | HF947463 | HE600686.1 | *Limnohabitans sp.* | 99 | | Betaproteobacteria |
| BF_T41 | HF947464 | JF710262.1 | *Chitinophaga sp.* | 91 | | Bacteriodetes |
| BF_T42 | HF947465 | JF710262.1 | *Chitinophaga sp.* | 91 | | Bacteriodetes |
| BF_T43 | HF947466 | GQ140629.1 | *Alistipes sp.* | 89 | | Bacteriodetes |
| BF_T44 | HF947467 | NR 025421.1 | *Limnobacter thiooxidans* | 91 | | Betaproteobacteria |
| BF_T45 | HF947468 | JF710262.1 | *Chitinophaga sp.* | 91 | | Bacteriodetes |
| BF_T46 | HF947469 | JF710262.1 | *Chitinophaga sp.* | 91 | | Bacteriodetes |
| BF_T47 | HF947470 | JF710262.1 | *Chitinophaga sp.* | 91 | | Bacteriodetes |
| BF_T48 | HF947471 | HE600686.1 | *Limnohabitans sp.* | 99 | | Betaproteobacteria |
| BF_T49 | HF947472 | JF710262.1 | *Chitinophaga sp.* | 91 | | Bacteriodetes |
| BF_T50 | HF947473 | JF710262.1 | *Chitinophaga sp.* | 91 | | Bacteriodetes |
| BF_T51 | HF947474 | AB353123.1 | *Cetobacterium somerae* | 96 | | Fusobacteriales |
| BF_T52 | HF947475 | JF710262.1 | *Chitinophaga sp.* | 91 | | Bacteriodetes |
| BF_T53 | HF947476 | JF710262.1 | *Chitinophaga sp.* | 91 | | Bacteriodetes |
| BF_T54 | HF947477 | JF710262.1 | *Chitinophaga sp.* | 91 | | Bacteriodetes |
| BF_T55 | HF947478 | JF710262.1 | *Chitinophaga sp.* | 91 | | Bacteriodetes |
| BF_T56 | HF947479 | JF710262.1 | *Chitinophaga sp.* | 91 | | Bacteriodetes |
| BF_T57 | HF947480 | NR 029213.2 | *Burkholderia graminis* | 85 | | Betaproteobacteria |
| BF_T58 | HF947481 | JF710262.1 | *Chitinophaga sp.* | 92 | | Bacteriodetes |
| BF_T59 | HF947482 | AB688628.1 | *Rickettsiaceae endo-symbiont of carteriacerasiformes* | 100 | | Alphaproteobacteria |
| BF_T60 | HF947483 | NR 025421.1 | *Limnobacter thiooxidans* | 90 | | Betaproteobacteria |
| BF_T61 | HF947484 | JF710262.1 | *Chitinophaga sp.* | 91 | | Bacteriodetes |
| BF_T62 | HF947485 | JF710262.1 | *Chitinophaga sp.* | 91 | | Bacteriodetes |
| BF_T63 | HF947486 | JF824804.1 | *Alistipes sp.* | 89 | | Bacteriodetes |
| BF_T64 | HF947487 | JF710262.1 | *Chitinophaga sp.* | 91 | | Bacteriodetes |
| BF_T65 | HF947488 | JF710262.1 | *Chitinophaga sp.* | 91 | | Bacteriodetes |
| BF_T66 | HF947489 | JF710262.1 | *Chitinophaga sp.* | 91 | | Bacteriodetes |
| BF_T67 | HF947490 | JF710262.1 | *Chitinophaga sp.* | 90 | | Bacteriodetes |
| BF_T68 | HF947491 | JF710262.1 | *Chitinophaga sp.* | 91 | | Bacteriodetes |
| BF_T69 | HF947492 | NR 025421.1 | *Limnobacter thiooxidans* | 91 | | Betaproteobacteria |
| BF_T70 | HF947493 | JF710262.1 | *Chitinophaga sp.* | 91 | | Bacteriodetes |
| BF_T72 | HF947494 | HE600686.1 | *Limnohabitans sp.* | 99 | | Betaproteobacteria |
| BF_T74 | HF947495 | AB353123.1 | *Cetobacterium somerae* | 97 | | Fusobacteriales |
| BF_T76 | HF947496 | JF710262.1 | *Chitinophaga sp.* | 91 | | Bacteriodetes |
| BF_T77 | HF947497 | JF710262.1 | *Chitinophaga sp.* | 90 | | Bacteriodetes |
| BF_T78 | HF947498 | JF710262.1 | *Chitinophaga sp.* | 90 | | Bacteriodetes |
| BF_T79 | HF947499 | HE600686.1 | *Limnohabitans sp.* | 99 | | Betaproteobacteria |
| BF_T80 | HF947500 | NR 025421.1 | *Limnobacter thiooxidans* | 91 | | Betaproteobacteria |
| BF_T82 | HF947501 | JF824804.1 | *Alistipes sp.* | 89 | | Bacteriodetes |
| BF_T83 | HF947502 | JF710262.1 | *Chitinophaga sp.* | 90 | | Bacteriodetes |
| BF_T84 | HF947503 | JF710262.1 | *Chitinophaga sp.* | 90 | | Bacteriodetes |
| BF_T85 | HF947504 | JF710262.1 | *Chitinophaga sp.* | 90 | | Bacteriodetes |
| BF_T86 | HF947505 | AB360415.1 | *Niastella sp.* | 92 | | Bacteriodetes |
| BF_T88 | HF947506 | JQ317253.1 | *Bacteriodes sp.* | 91 | | Bacteriodetes |
| BF_T89 | HF947507 | AB360415.1 | *Niastella sp.* | 91 | | Bacteriodetes |
| BF_T95 | HF947508 | NR 025421.1 | *Limnobacter thiooxidans* | 91 | | Betaproteobacteria |
| BF_T96 | HF947509 | JF710262.1 | *Chitinophaga sp.* | 91 | | Bacteriodetes |
